# Supplementary material for: Paw pressure and gait in middle-aged client-owned cats with and without naturally-occurring musculoskeletal disease
Source: PLoS One. 2024 Dec 18;19(12):e0314629. doi: 10.1371/journal.pone.0314629 (PMC11654939; doi:10.1371/journal.pone.0314629)
Supplement: S1 Table — Min = minimum value, Max = maximum value, Q1 = 1st quartile, Q3 = 3rd quartile, SD = standard deviation. Values in bold indicate distribution. (DOCX) [file pone.0314629.s001.docx]

| **Supplementary data Table 1:** Summary of co-efficient of variation in gait parameters from n-=31 apparently healthy middle-aged cats | | | | | | | |
| --- | --- | --- | --- | --- | --- | --- | --- |
|  | Mean | Median | Min | Q1 | Q3 | Max | SD |
| *Velocity (m/sec)* | 17.37 | **17.48** | 4.50 | **14.68** | **20.23** | 30.28 | 5.457 |
| *Gait cycle time (sec)* | **13.03** | 13.17 | **5.62** | 10.43 | 16.01 | **19.69** | **3.613** |
| ***Left Forelimb*** |  |  |  |  |  |  |  |
| *Stance Time (sec)* | 13.22 | **14.25** | 0.00 | **10.31** | **16.54** | 22.11 | 5.211 |
| *Swing Time (sec)* | 7.37 | **7.44** | 0.00 | **5.88** | **8.80** | 14.94 | 3.256 |
| *Stride Length (m)* | 4.68 | **4.88** | 0.69 | **2.72** | **6.63** | 8.26 | 2.197 |
| *Stride Velocity (m/sec)* | 14.41 | **14.76** | 3.20 | **11.32** | **18.15** | 23.27 | 5.148 |
| *Peak Vertical Force (N)* | **15.86** | 14.20 | **4.52** | 10.97 | 20.62 | **37.63** | **7.133** |
| *Vertical Impulse (Ns)* | **18.46** | 18.33 | **6.61** | 13.59 | 20.74 | **34.35** | **7.044** |
| *Force normalised to mass* | **10.03** | 8.09 | **3.21** | 7.01 | 13.36 | **20.10** | **4.536** |
| *Impulse normalised to mass* | **15.40** | 18.82 | **4.16** | 12.79 | 18.33 | **30.69** | **4.915** |
| ***Right Forelimb*** |  |  |  |  |  |  |  |
| *Stance Time (sec)* | 13.43 | **14.69** | 1.43 | **8.68** | **18.39** | 24.79 | 6.531 |
| *Swing Time (sec)* | 9.87 | **8.89** | 0.00 | **6.68** | **12.80** | 20.74 | 4.698 |
| *Stride Length (m)* | 4.82 | **4.25** | 0.77 | **3.35** | **6.50** | 8.17 | 2.059 |
| *Stride Velocity (m/sec)* | 14.60 | **17.22** | 0.00 | **8.61** | **20.07** | 27.91 | 7.705 |
| *Peak Vertical Force (N)* | **15.82** | 15.46 | 8.12 | 11.65 | 18.59 | **29.79** | **5.377** |
| *Vertical Impulse (Ns)* | **17.34** | 15.99 | 7.29 | 13.55 | 19.84 | **32.84** | **6.130** |
| *Force normalised to mass* | **9.88** | 9.26 | **6.09** | 7.609 | 12.06 | **15.38** | **2.806** |
| *Impulse normalised to mass* | **14.27** | 13.81 | **3.79** | 11.06 | 17.69 | **27.05** | **4.958** |
| ***Left Hindlimb*** |  |  |  |  |  |  |  |
| *Stance Time (sec)* | 13.41 | **13.07** | 0.00 | **8.19** | **17.02** | 36.81 | 8.071 |
| *Swing Time (sec)* | 10.23 | **9.12** | 0.00 | **7.10** | **13.46** | 21.21 | 4.837 |
| *Stride Length (m)* | 5.11 | **4.92** | 0.78 | **3.46** | **6.85** | 11.68 | 2.779 |
| *Stride Velocity (m/sec)* | 16.68 | **17.84** | 2.82 | **12.03** | **21.52** | 33.65 | 7.225 |
| *Peak Vertical Force (N)* | **15.19** | 14.89 | **5.63** | 11.49 | 18.44 | **26.73** | **5.125** |
| *Vertical Impulse (Ns)* | **21.76** | 19.88 | **6.61** | 16.06 | 25.53 | **53.69** | **8.880** |
| *Force normalised to mass* | **11.79** | 11.86 | **5.52** | 8.81 | 14.74 | **20.41** | **3.969** |
| *Impulse normalised to mass* | **16.56** | 15.29 | **7.42** | 12.19 | 19.23 | **38.31** | **6.081** |
| ***Right Hindlimb*** |  |  |  |  |  |  |  |
| *Stance Time (sec)* | 14.01 | **14.35** | 4.56 | **10.34** | **17.54** | 26.17 | 5.720 |
| *Swing Time (sec)* | 10.17 | **8.91** | 0.00 | **6.98** | **13.58** | 20.20 | 4.797 |
| *Stride Length (m)* | 4.32 | **4.02** | 0.00 | **2.36** | **6.24** | 10.68 | 2.722 |
| *Stride Velocity (m/sec)* | 15.21 | **16.02** | 0.93 | **11.17** | **20.02** | 25.85 | 6.713 |
| *Peak Vertical Force (N)* | **15.31** | 14.67 | **4.82** | 12.06 | 18.92 | **24.94** | **5.290** |
| *Vertical Impulse (Ns)* | **21.83** | 20.37 | **11.33** | 16.05 | 26.34 | **44.64** | **7.834** |
| *Force normalised to mass* | **11.15** | 10.91 | **4.04** | 8.83 | 13.58 | **20.60** | **3.798** |
| *Impulse normalised to mass* | **16.67** | 16.21 | **6.45** | 13.20 | 19.29 | **30.51** | **4.803** |
| Min = minimum value, Max = maximum value, Q1 = 1^st^ quartile, Q3 = 3^rd^ quartile, SD = standard deviation. Values in bold indicate distribution. | | | | | | | |
