# Supplementary material for: Paw pressure and gait in middle-aged client-owned cats with and without naturally-occurring musculoskeletal disease
Source: PLoS One. 2024 Dec 18;19(12):e0314629. doi: 10.1371/journal.pone.0314629 (PMC11654939; doi:10.1371/journal.pone.0314629)
Supplement: S2 Table — Min = minimum value, Max = maximum value, Q1 = 1st quartile, Q3 = 3rd quartile, SD = standard deviation. Values in bold indicate distribution. (DOCX) [file pone.0314629.s002.docx]

| **Supplementary data Table 2:** Summary of co-efficient of variation in gait parameter from n=22 middle-aged cats with musculoskeletal disease | | | | | | | |
| --- | --- | --- | --- | --- | --- | --- | --- |
|  | Mean | Median | Min | Q1 | Q3 | Max | SD |
| *Velocity (m/sec)* | 18.88 | **17.99** | 10.32 | **14.50** | **22.29** | 33.11 | 6.039 |
| *Gait cycle time (sec)* | **14.96** | 14.55 | **6.98** | 11.93 | 17.14 | **23.75** | **4.708** |
| ***Left Forelimb*** |  |  |  |  |  |  |  |
| *Stance Time (sec)* | 16.92 | **16.03** | 3.24 | **12.00** | **20.99** | 32.86 | 7.563 |
| *Swing Time (sec)* | 10.75 | **8.98** | 0.00 | **5.96** | **14.13** | 26.05 | 6.587 |
| *Stride Length (m)* | 5.82 | **4.64** | 0.71 | **3.35** | **6.97** | 21.58 | 4.705 |
| *Stride Velocity (m/sec)* | 19.26 | **16.86** | 4.21 | **12.62** | **22.98** | 42.37 | 9.974 |
| *Peak Vertical Force (N)* | **14.83** | 13.66 | **1.96** | 11.80 | 17.43 | **31.13** | **5.963** |
| *Vertical Impulse (Ns)* | **18.46** | 17.33 | **7.44** | 11.68 | 24.03 | **33.82** | **7.480** |
| *Force normalised to mass* | **9.57** | 8.68 | **3.99** | 7.63 | 10.48 | **22.60** | **4.028** |
| *Impulse normalised to mass* | **16.05** | 14.49 | **6.61** | 12.53 | 18.46 | **32.87** | **6.10** |
| ***Right Forelimb*** |  |  |  |  |  |  |  |
| *Stance Time (sec)* | 15.86 | **14.61** | 5.81 | **11.83** | **18.76** | 30.50 | 6.463 |
| *Swing Time (sec)* | 8.05 | **6.59** | 0.00 | **3.74** | **12.20** | 22.63 | 6.091 |
| *Stride Length (m)* | 5.85 | **4.62** | 0.58 | **3.15** | **7.49** | 15.27 | 3.894 |
| *Stride Velocity (m/sec)* | 17.25 | **16.78** | 0.39 | **11.20** | **21.84** | 33.30 | 8.973 |
| *Peak Vertical Force (N)* | **14.82** | 13.69 | **5.56** | 10.65 | 16.96 | **33.07** | **6.537** |
| *Vertical Impulse (Ns)* | **16.33** | 15.83 | **7.42** | 12.31 | 19.25 | **29.75** | **5.982** |
| *Force normalised to mass* | **9.19** | 8.40 | **1.80** | 7.47 | 12.19 | **17.96** | **3.857** |
| *Impulse normalised to mass* | **14.80** | 15.28 | **7.08** | 10.29 | 17.99 | **27.78** | **5.284** |
| ***Left Hindlimb*** |  |  |  |  |  |  |  |
| *Stance Time (sec)* | 17.10 | **17.21** | 4.12 | **11.70** | **22.72** | 33.94 | 8.006 |
| *Swing Time (sec)* | 11.36 | **11.29** | 2.32 | **7.62** | **13.93** | 22.00 | 5.122 |
| *Stride Length (m)* | 4.73 | **4.04** | 0.59 | **2.92** | **5.69** | 12.43 | 3.165 |
| *Stride Velocity (m/sec)* | 18.45 | **18.21** | 3.27 | **12.37** | **22.54** | 44.11 | 9.811 |
| *Peak Vertical Force (N)* | **14.23** | 14.30 | **4.81** | 10.85 | 17.13 | **26.83** | **5.037** |
| *Vertical Impulse (Ns)* | **22.79** | 24.59 | **7.44** | 16.65 | 27.31 | **38.93** | **9.068** |
| *Force normalised to mass* | **9.35** | 8.92 | **2.47** | 6.79 | 12.69 | **15.31** | **3.786** |
| *Impulse normalised to mass* | **18.52** | 17.89 | **4.57** | 14.71 | 21.11 | **35.39** | **7.898** |
| ***Right Hindlimb*** |  |  |  |  |  |  |  |
| *Stance Time (sec)* | 15.27 | **13.95** | 4.29 | **8.01** | **21.69** | 31.43 | 8.199 |
| *Swing Time (sec)* | 10.45 | **10.43** | 2.24 | **5.83** | **13.20** | 28.93 | 6.367 |
| *Stride Length (m)* | 4.49 | **3.71** | 0.68 | **2.36** | **5.77** | 11.41 | 2.955 |
| *Stride Velocity (m/sec)* | 17.53 | **15.83** | 7.45 | **11.83** | **21.77** | 37.47 | 7.941 |
| *Peak Vertical Force (N)* | **14.79** | 15.22 | **7.96** | 12.46 | 16.22 | **23.97** | **4.298** |
| *Vertical Impulse (Ns)* | **20.59** | 19.78 | **6.75** | 16.34 | 24.97 | **33.89** | **7.011** |
| *Force normalised to mass* | **10.99** | 9.56 | **6.13** | 8.43 | 12.43 | **19.71** | **3.895** |
| *Impulse normalised to mass* | **16.12** | 16.27 | **7.98** | 13.53 | 19.17 | **20.06** | **3.91** |
| Min = minimum value, Max = maximum value, Q1 = 1^st^ quartile, Q3 = 3^rd^ quartile, SD = standard deviation. Values in bold indicate distribution. | | | | | | | |
