# Supplementary figures and images for: Paw pressure and gait in middle-aged client-owned cats with and without naturally-occurring musculoskeletal disease
Source: PLoS One. 2024 Dec 18;19(12):e0314629. doi: 10.1371/journal.pone.0314629 (PMC11654939; doi:10.1371/journal.pone.0314629)

**Supplementary Data 5: All Cat Data**

**Apparently healthy cats**


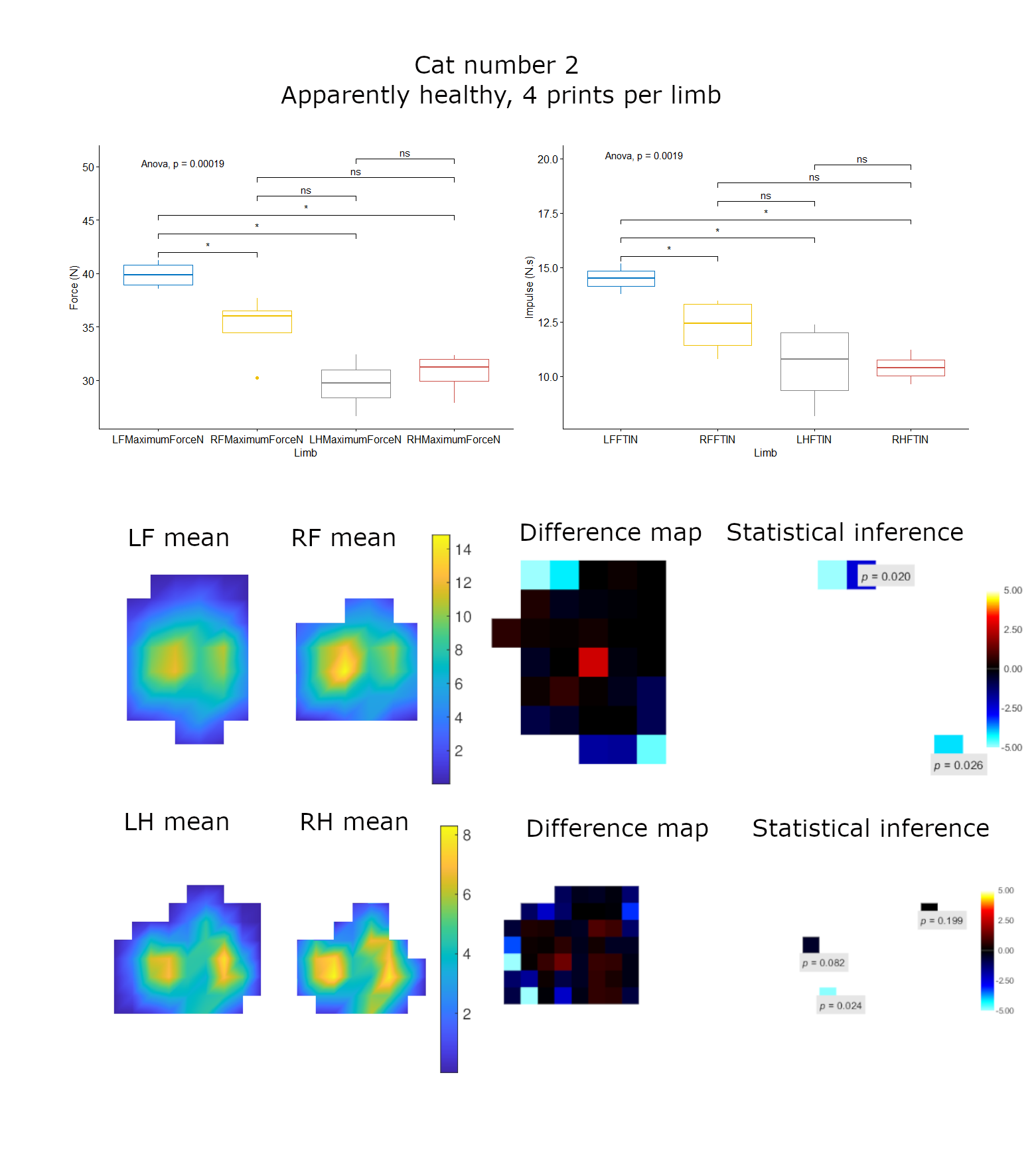


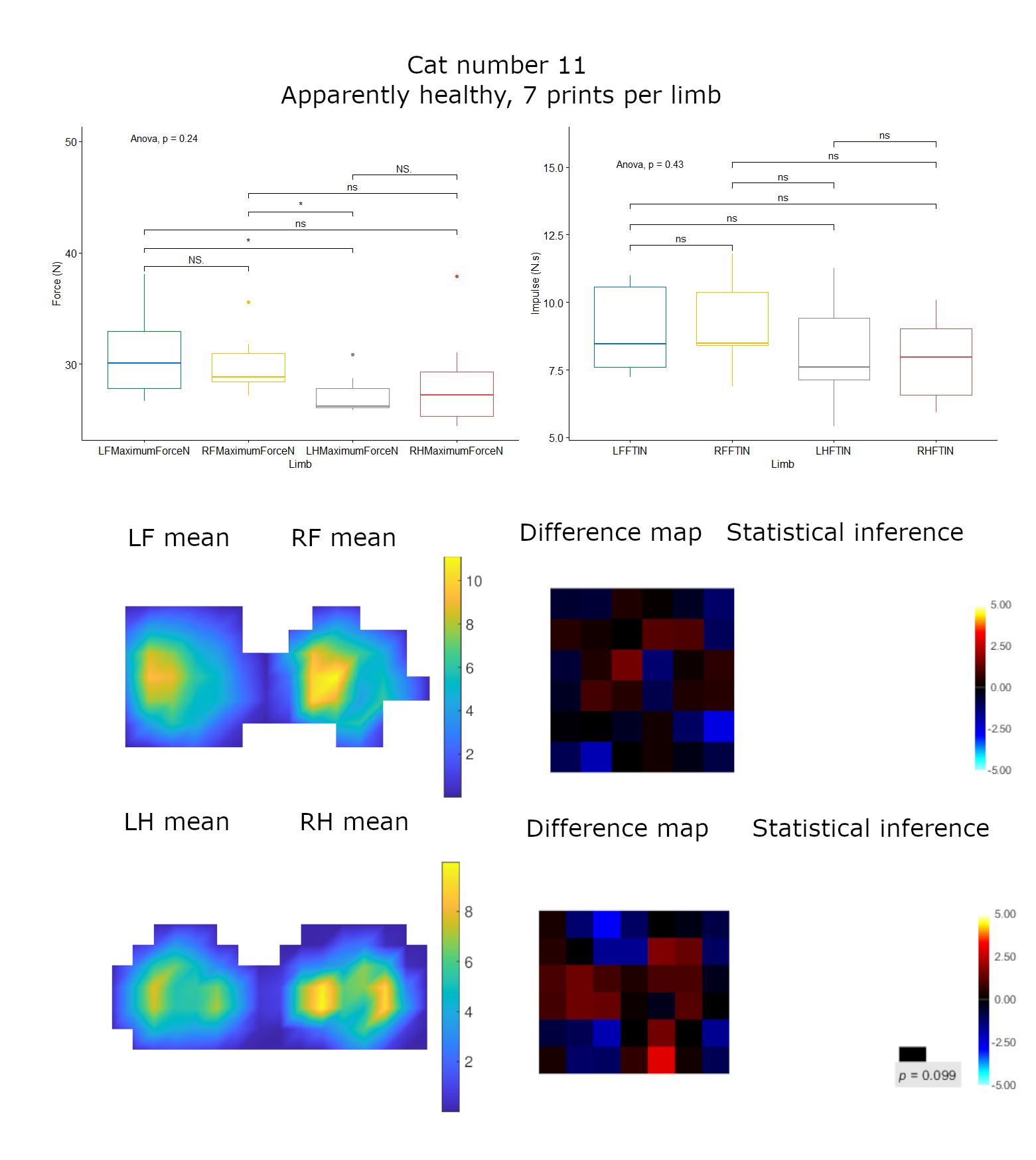


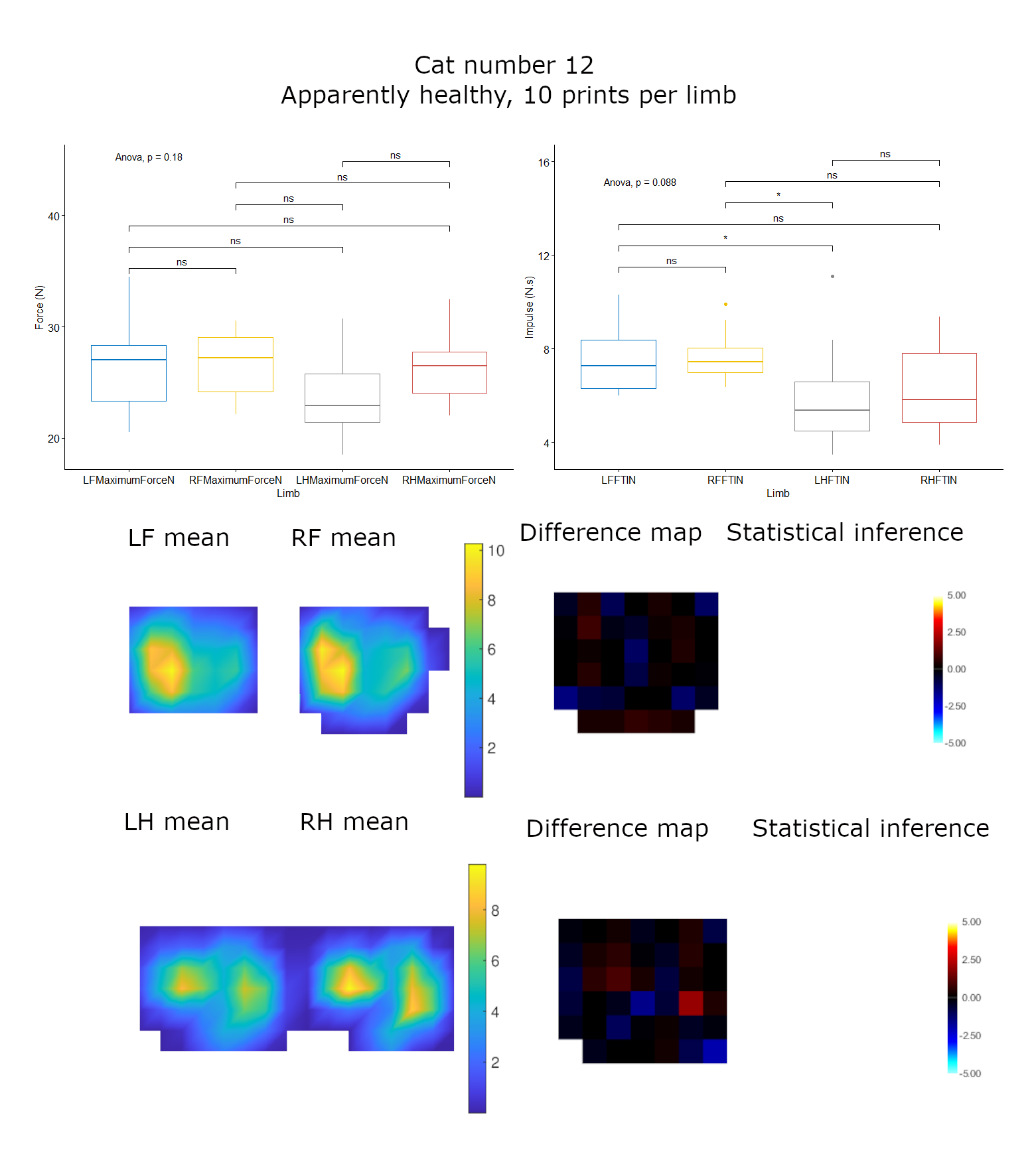

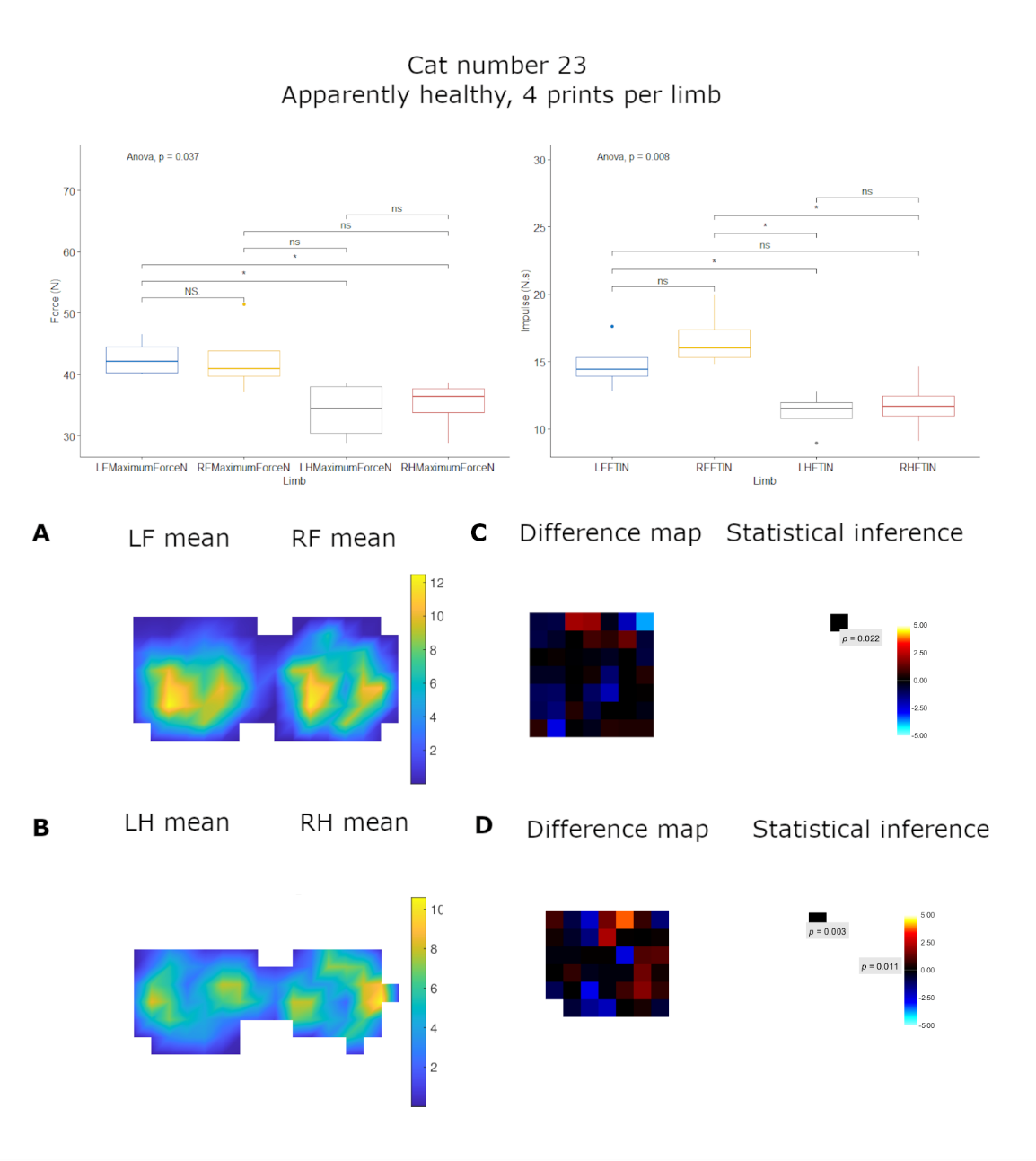

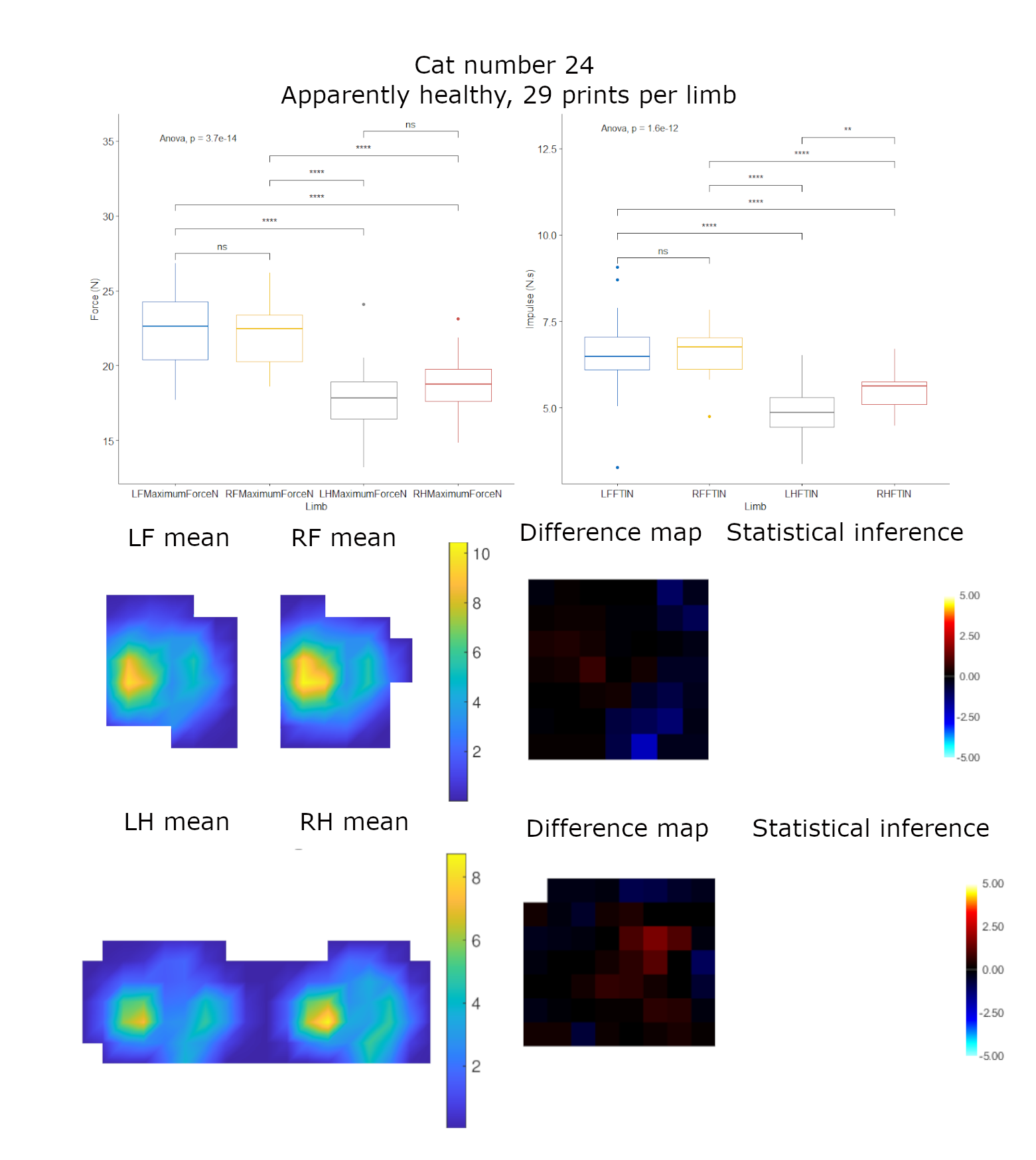

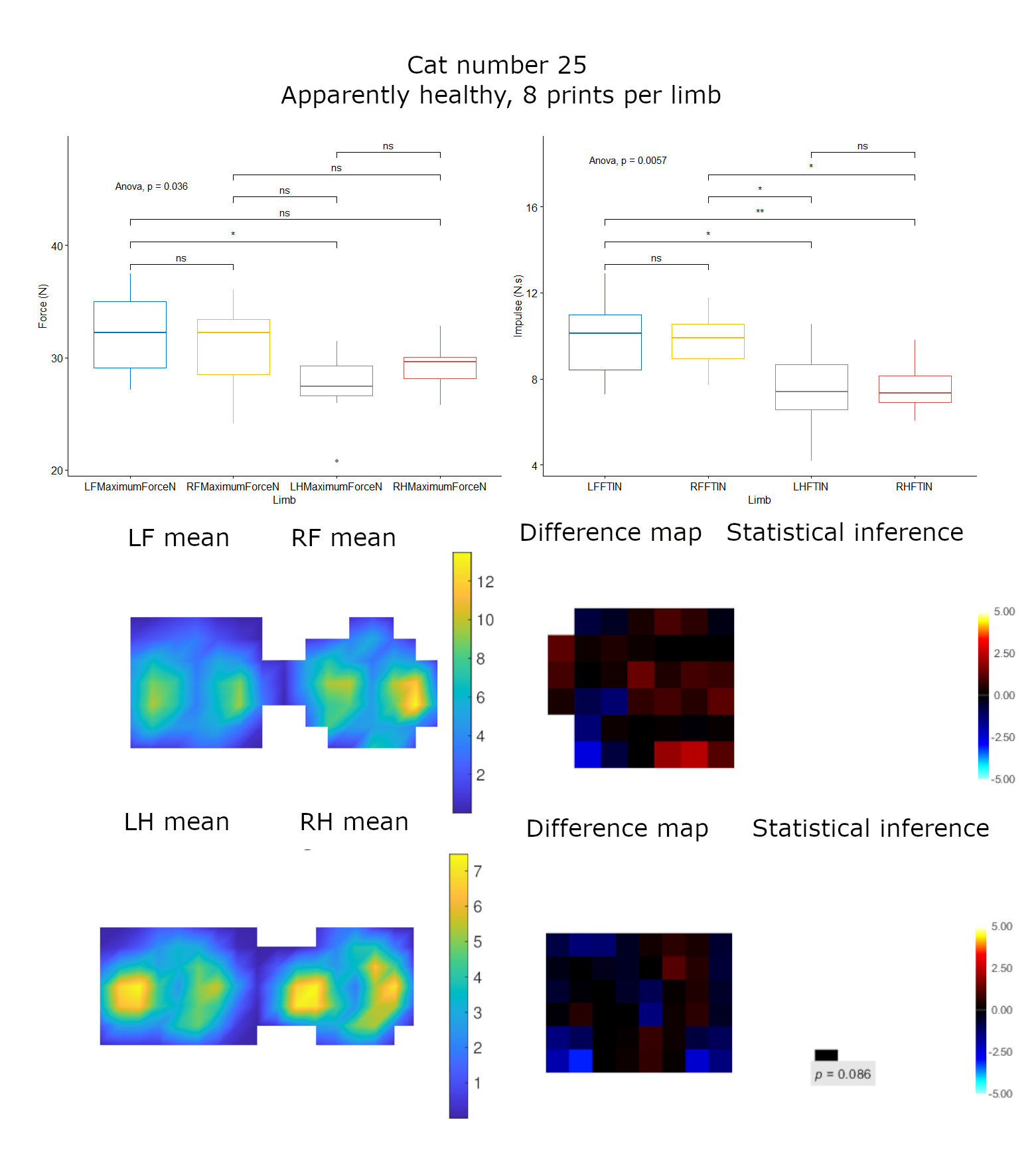


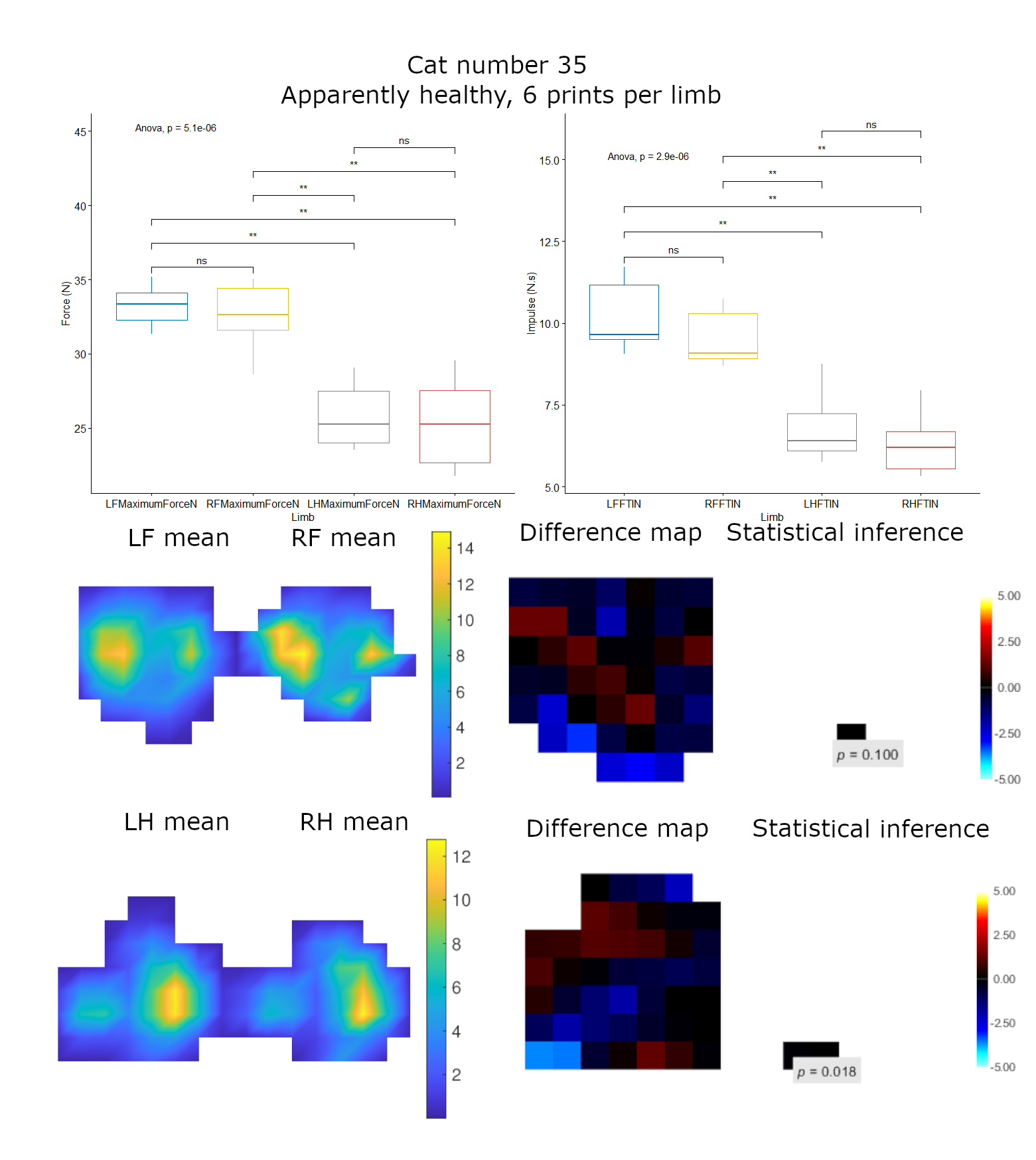

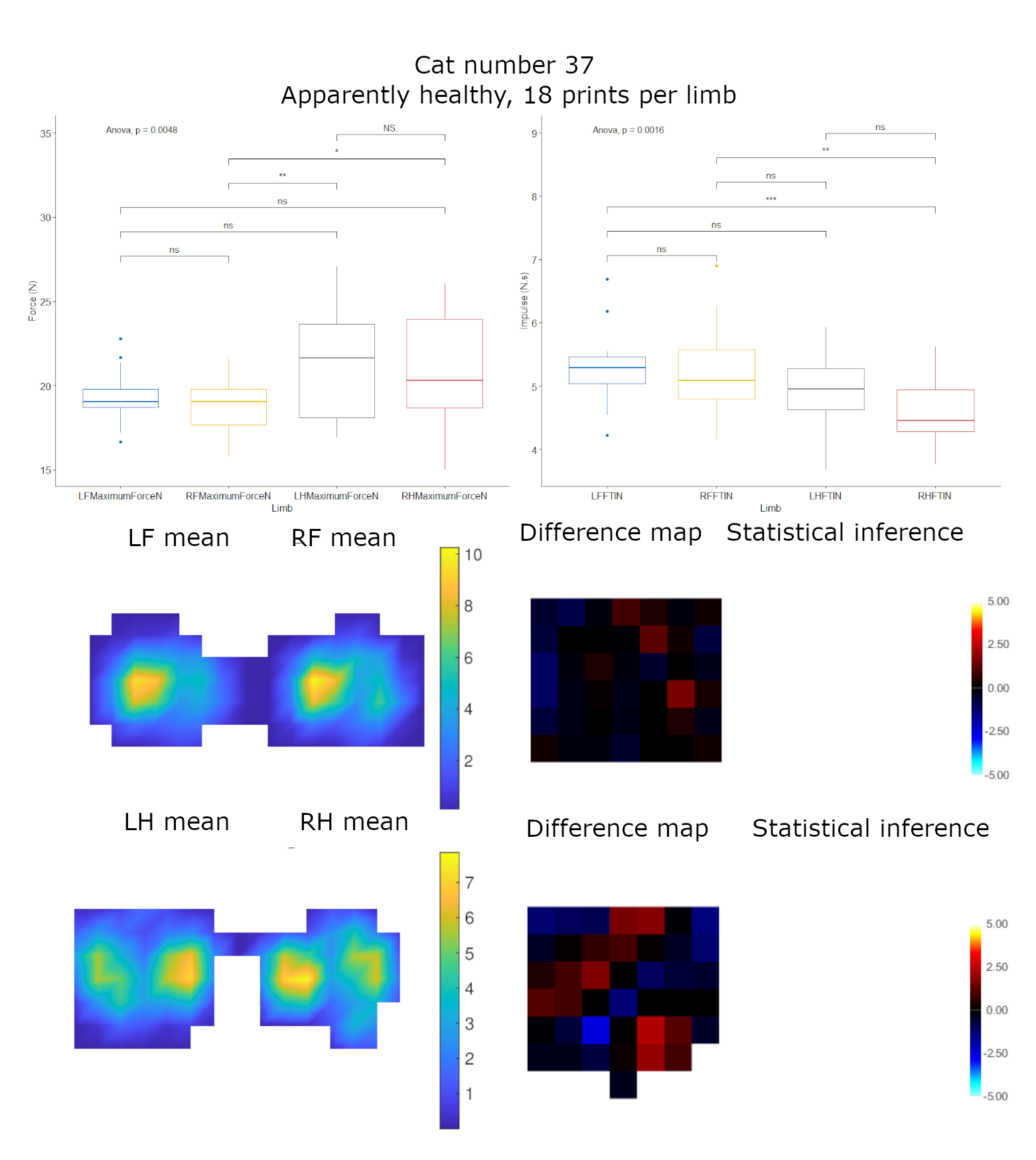


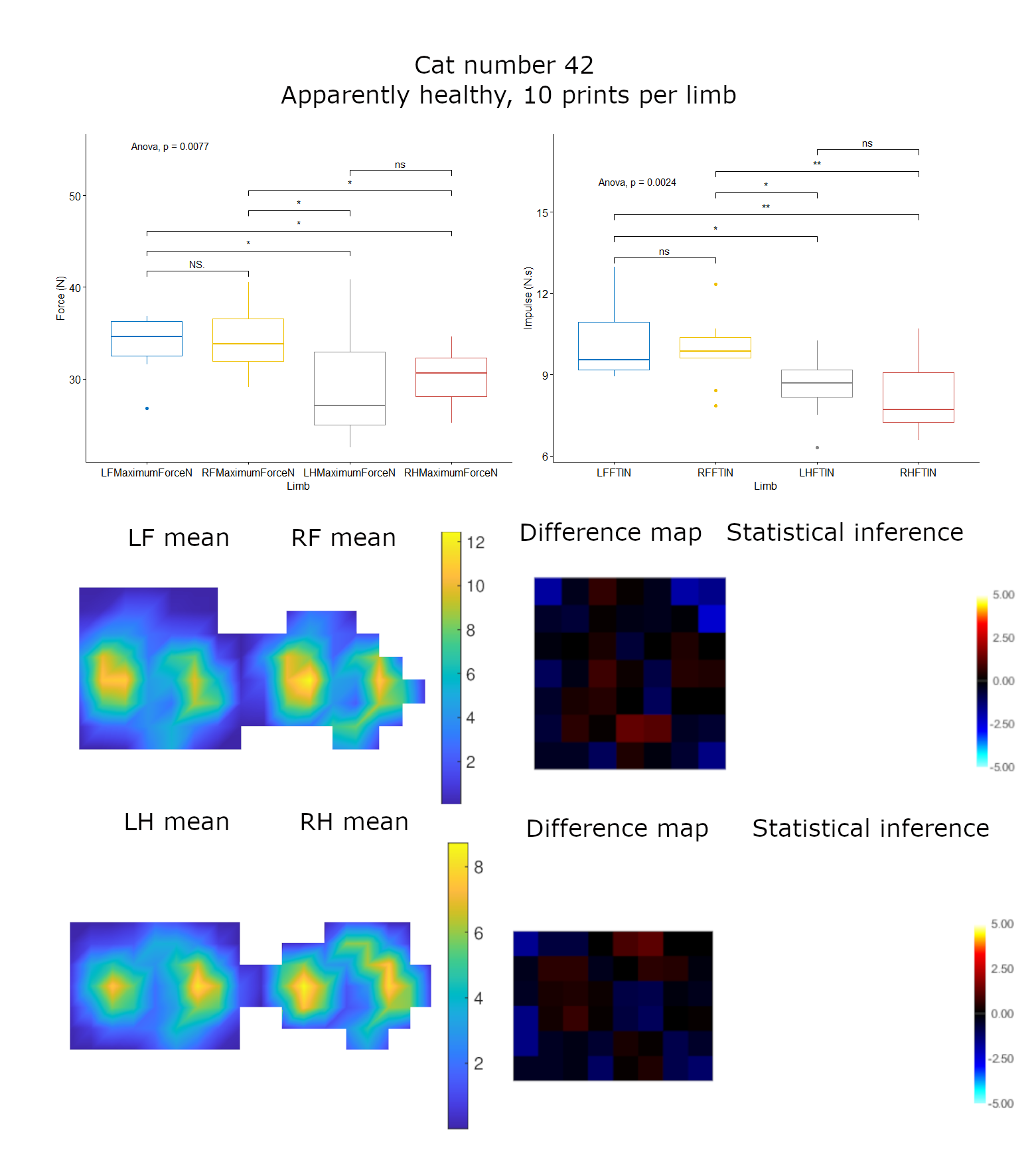

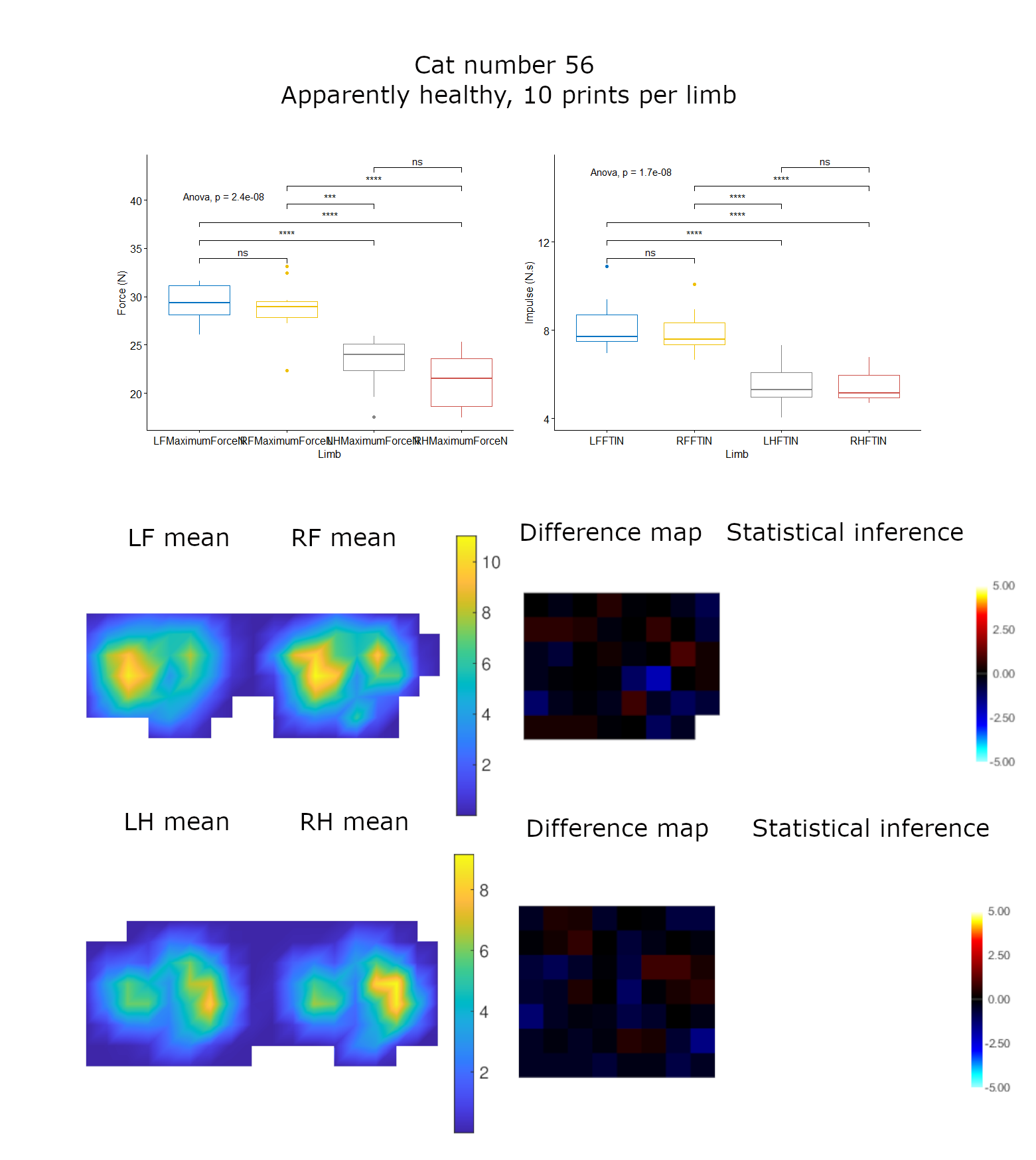


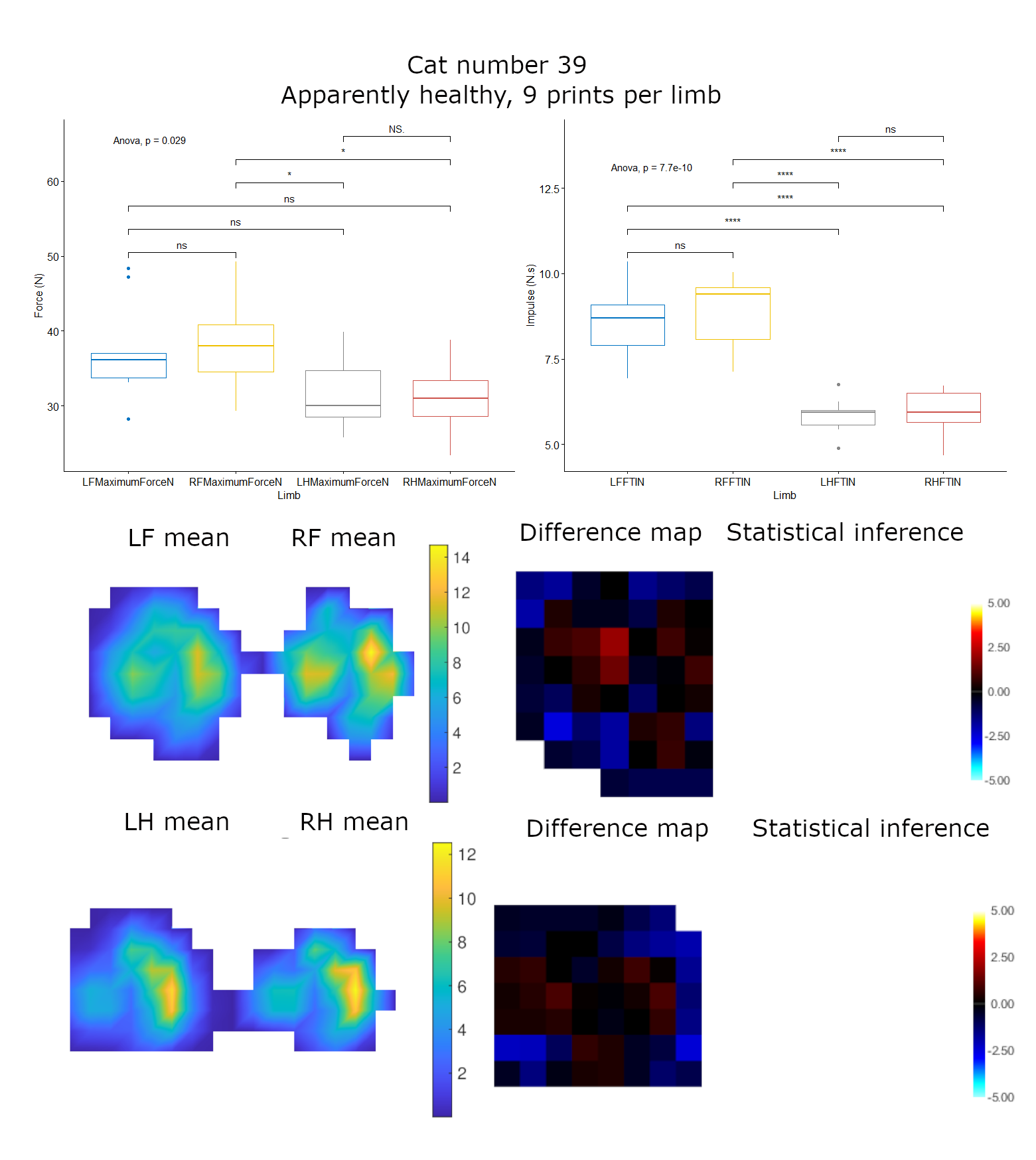

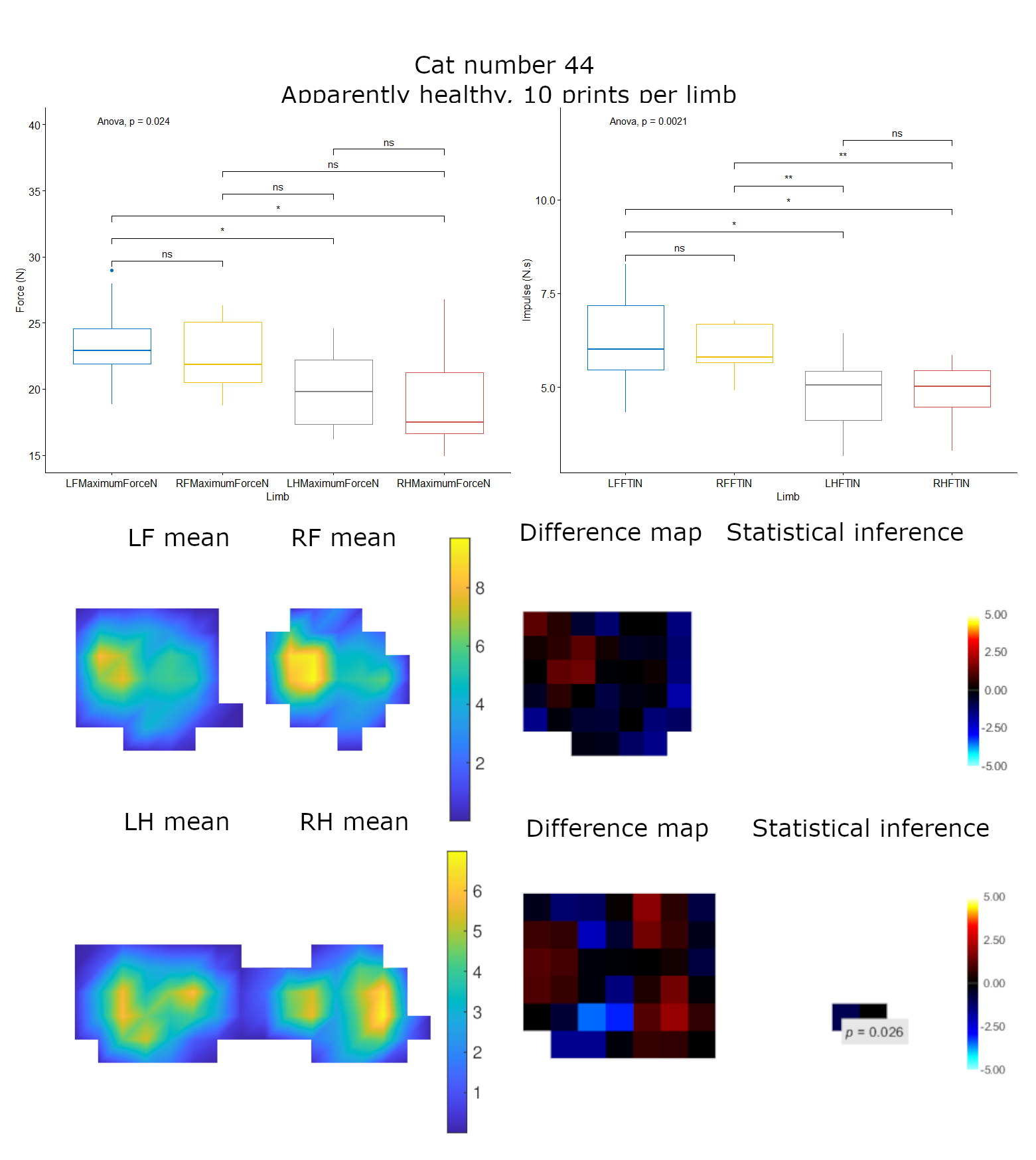

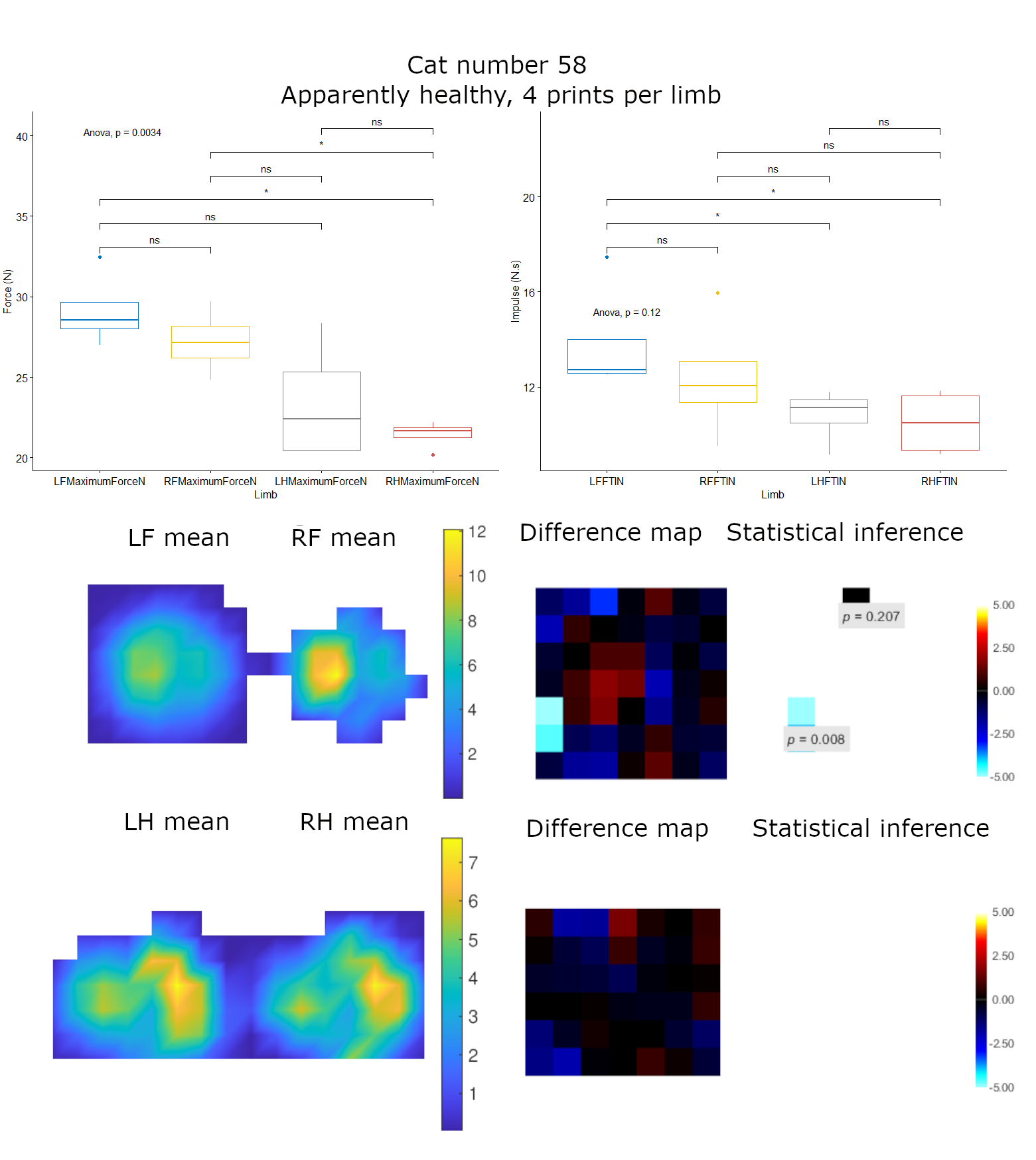

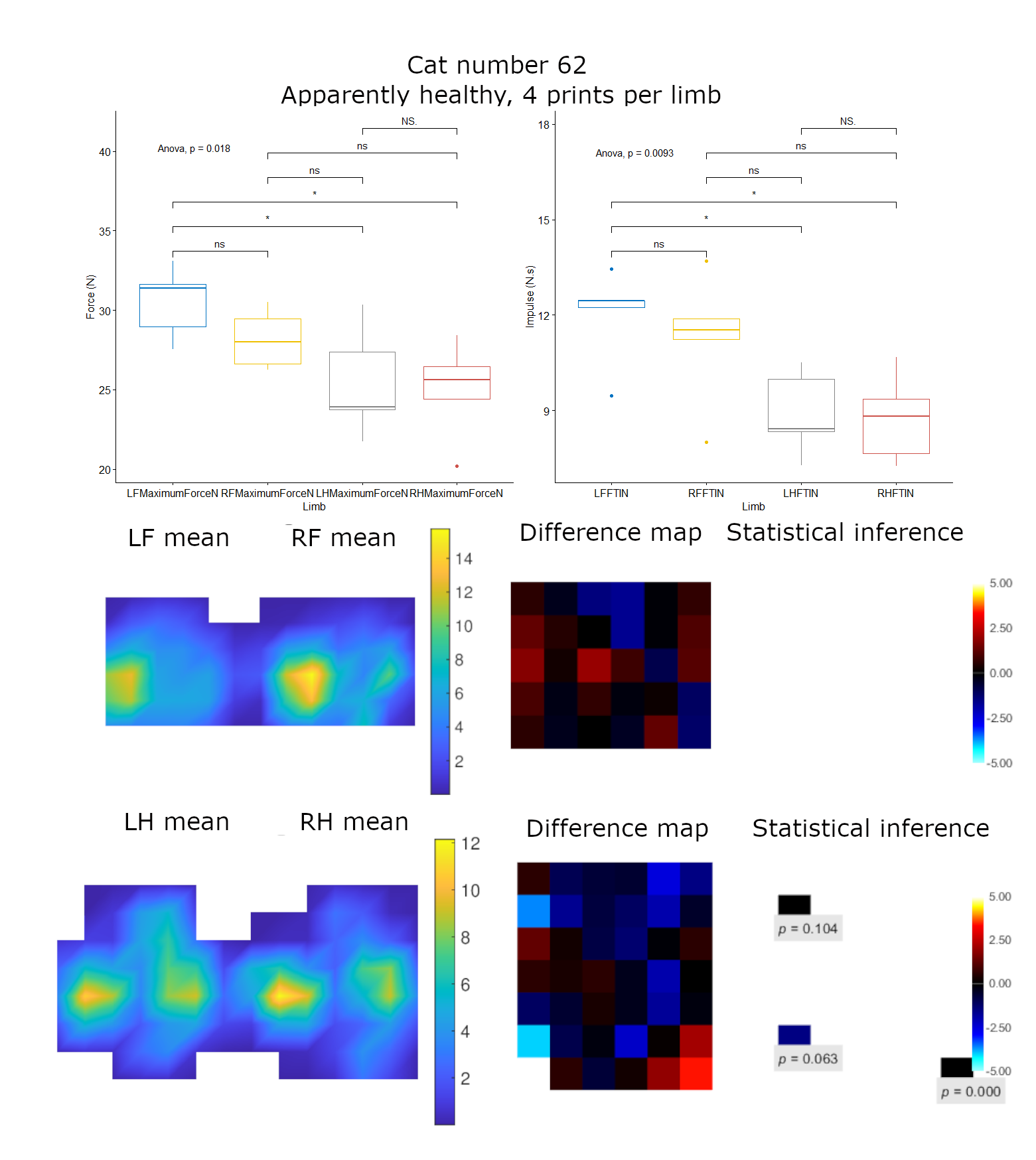

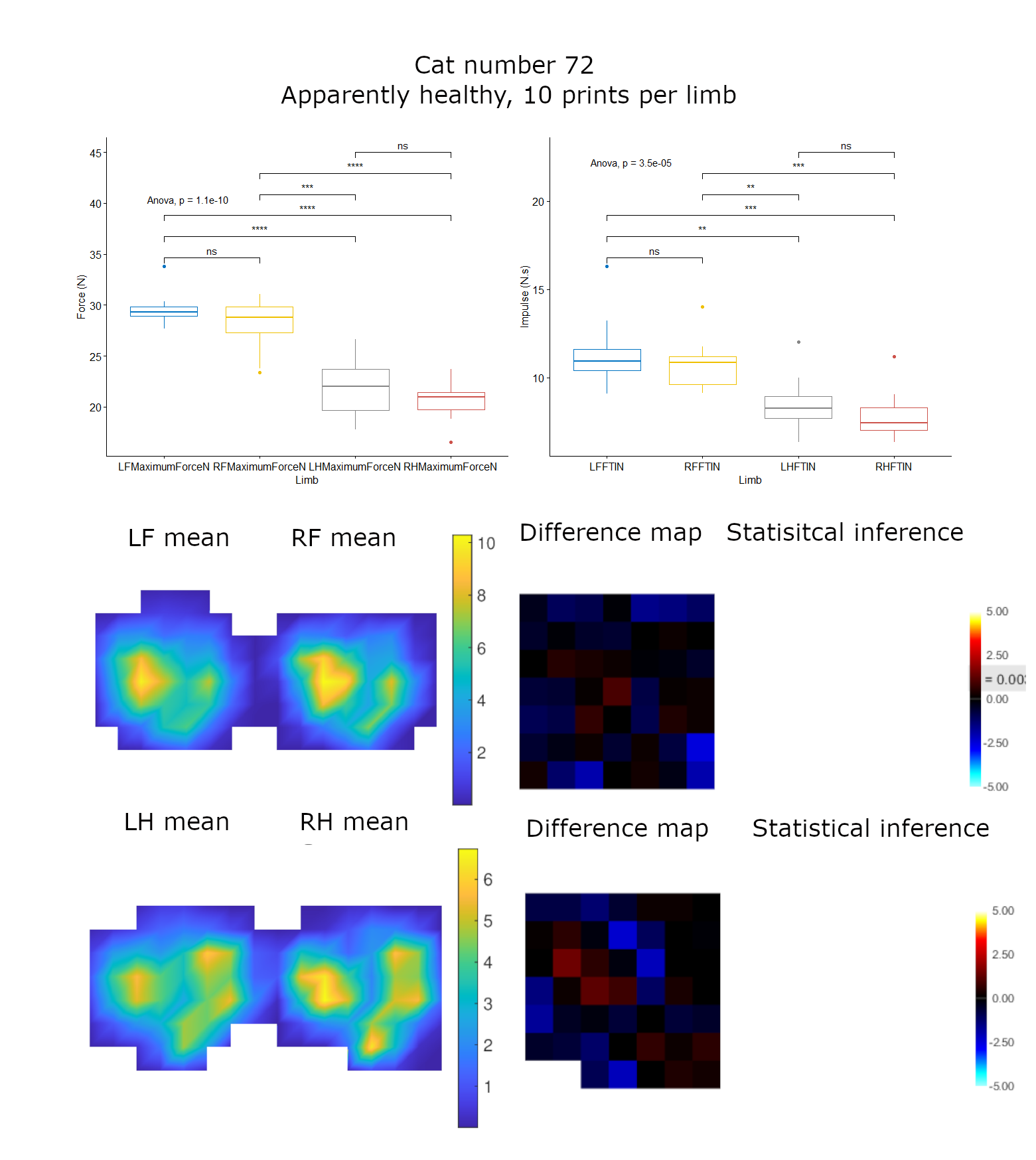

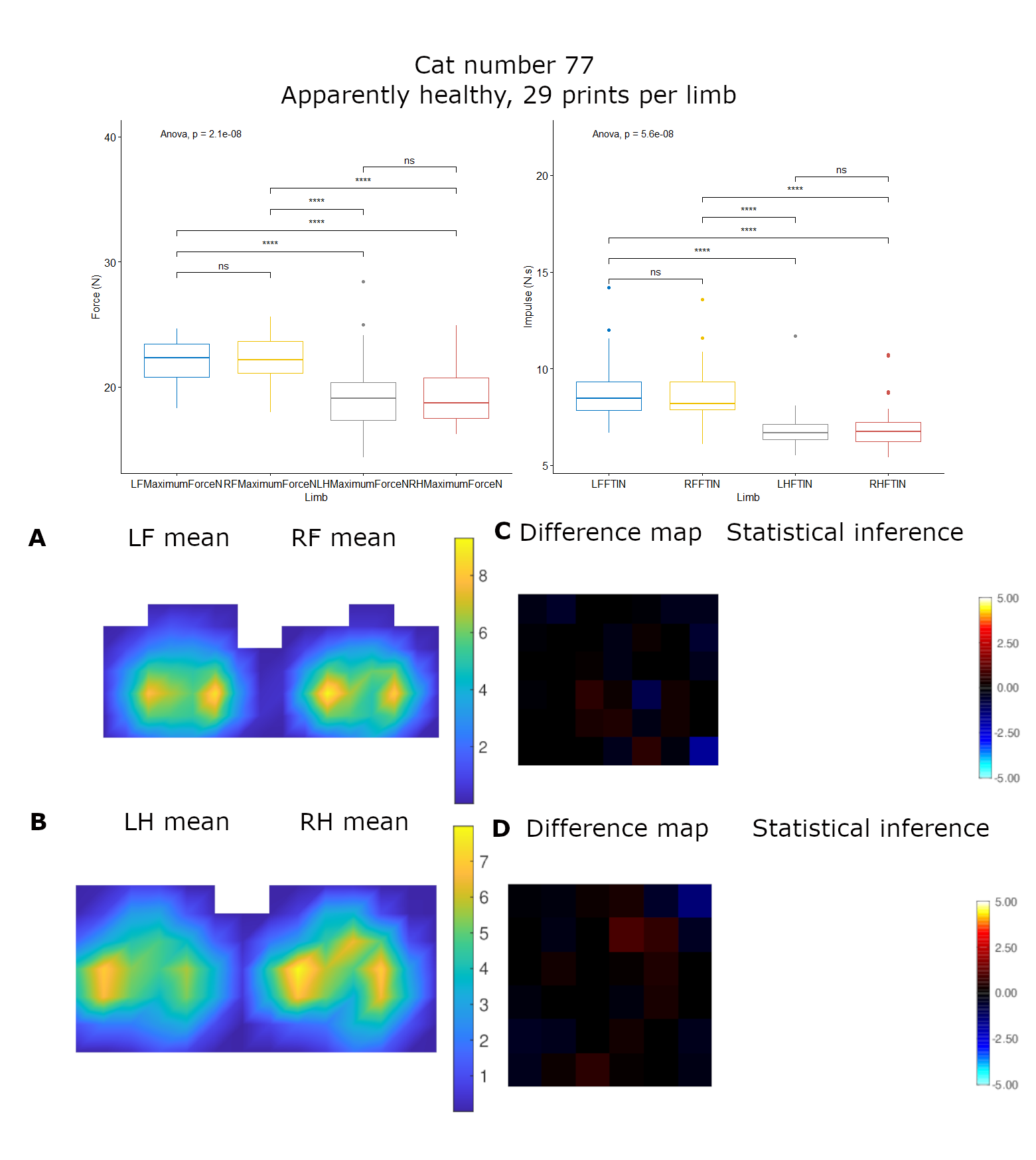

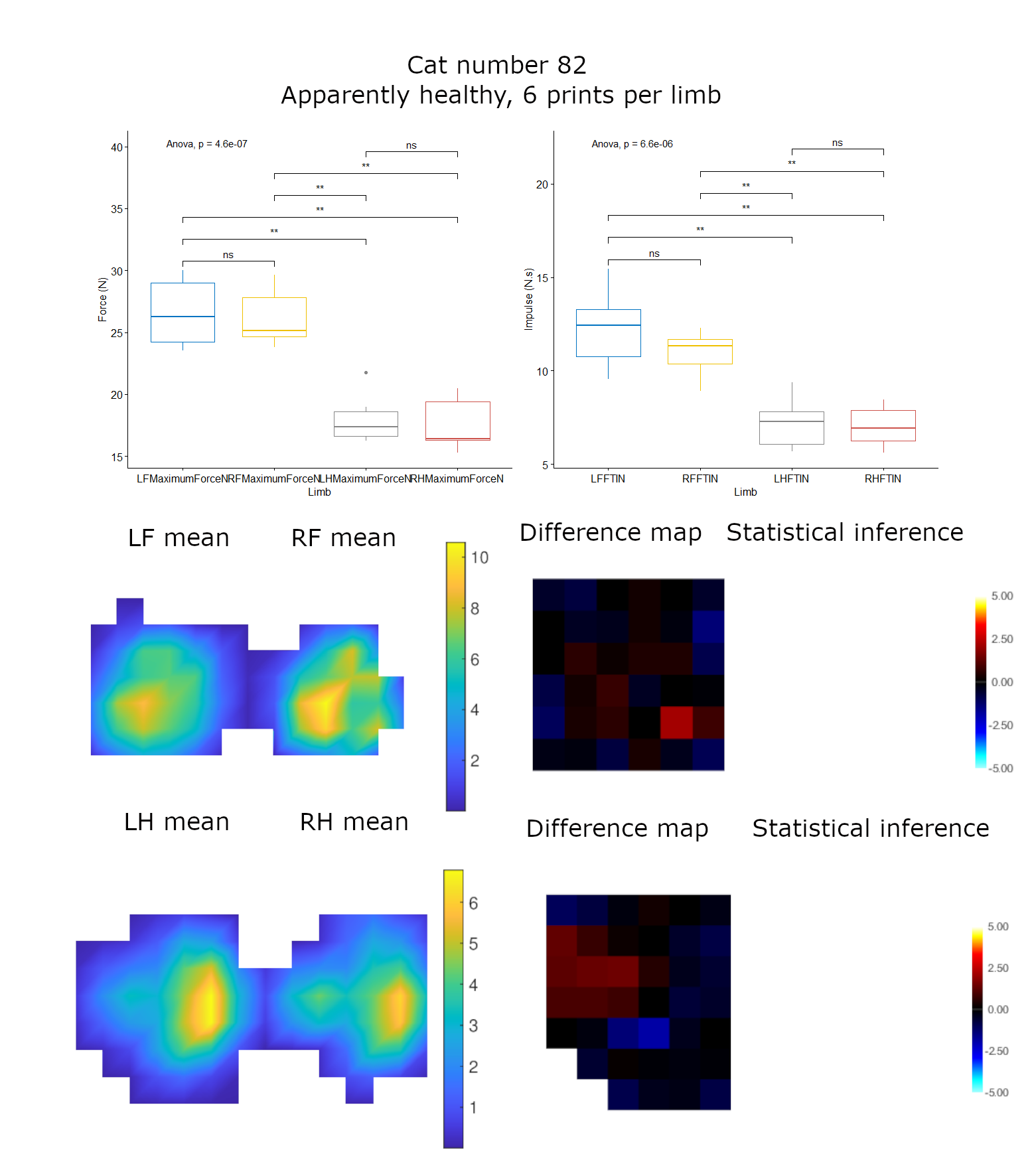

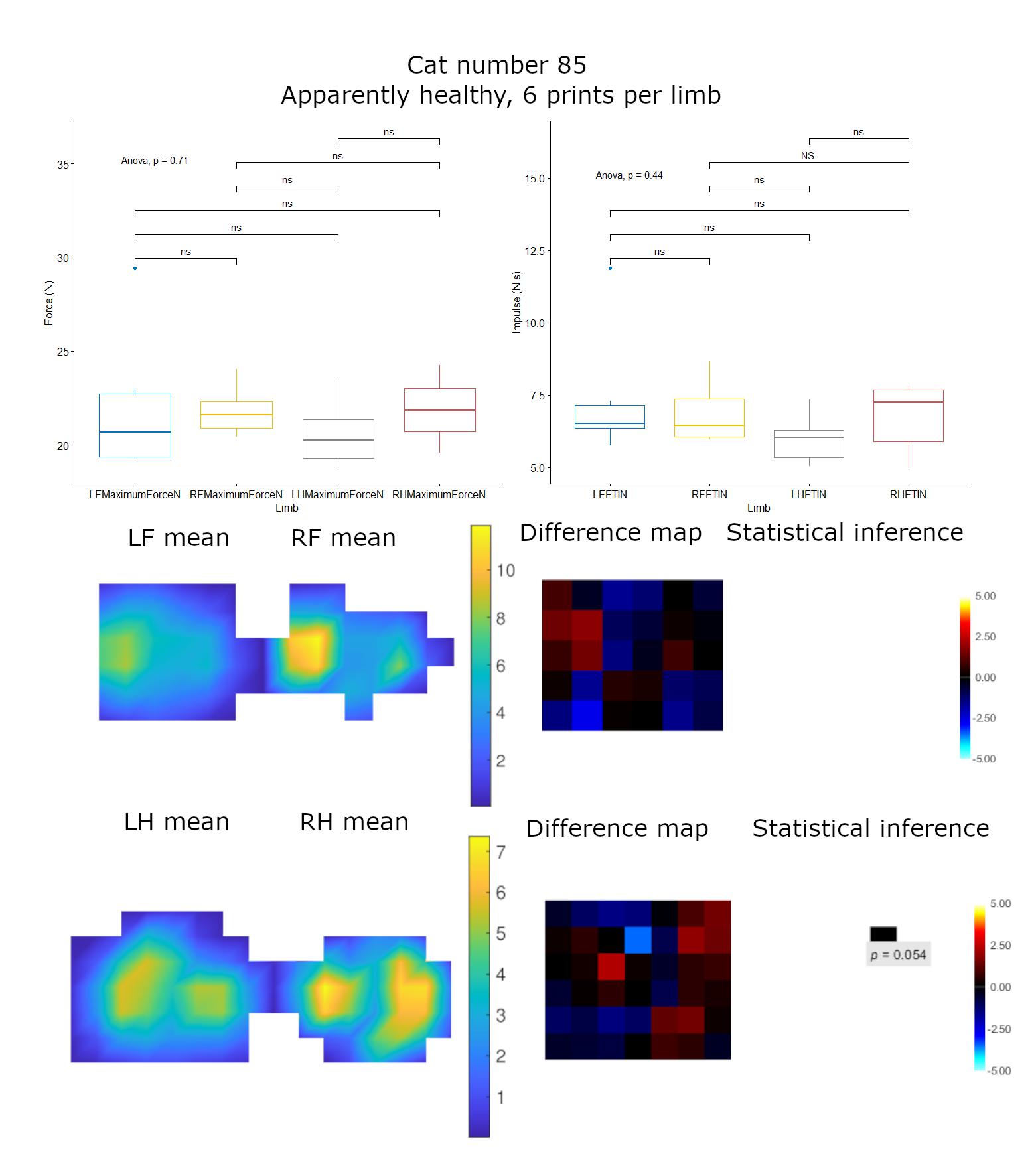

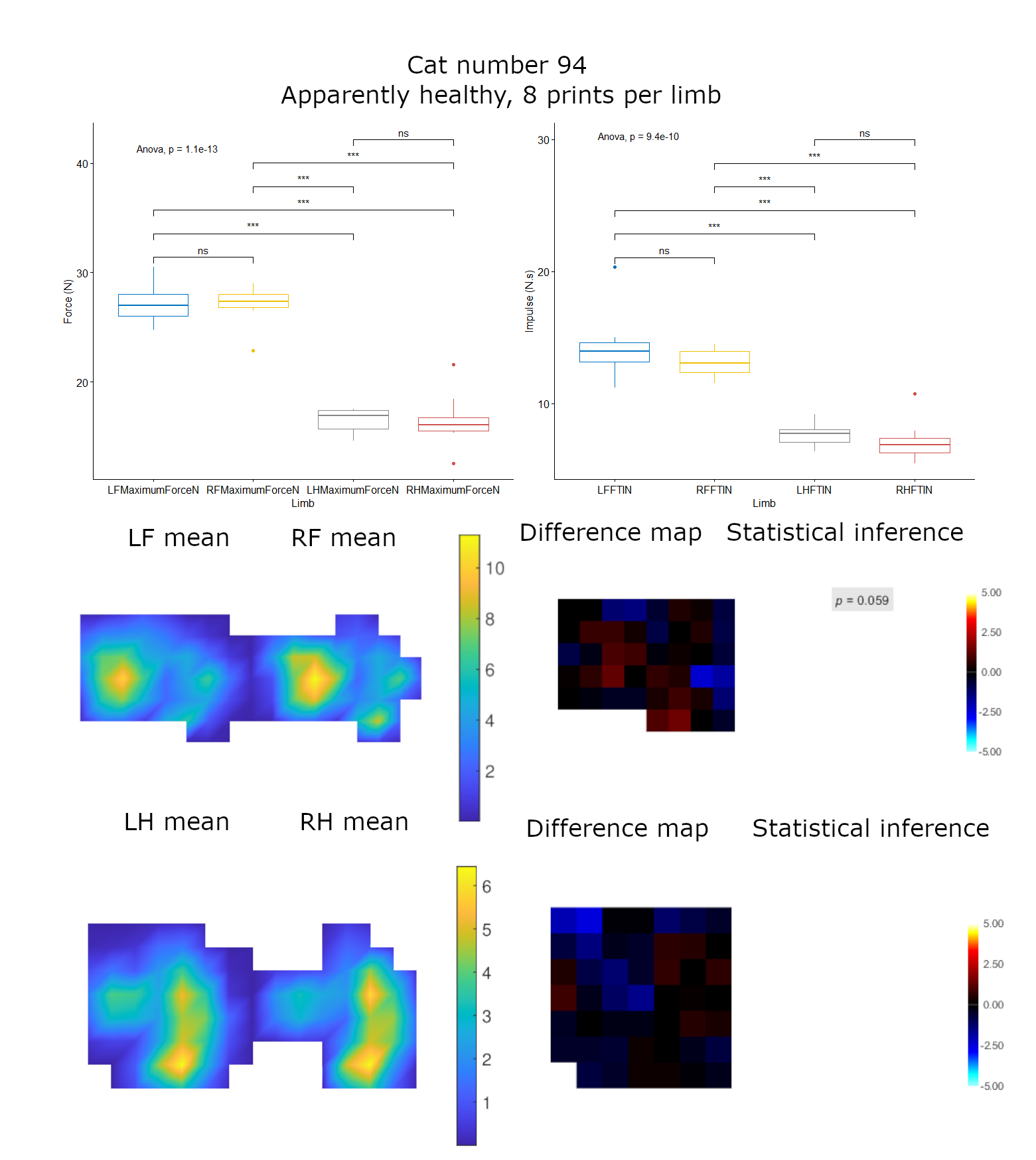

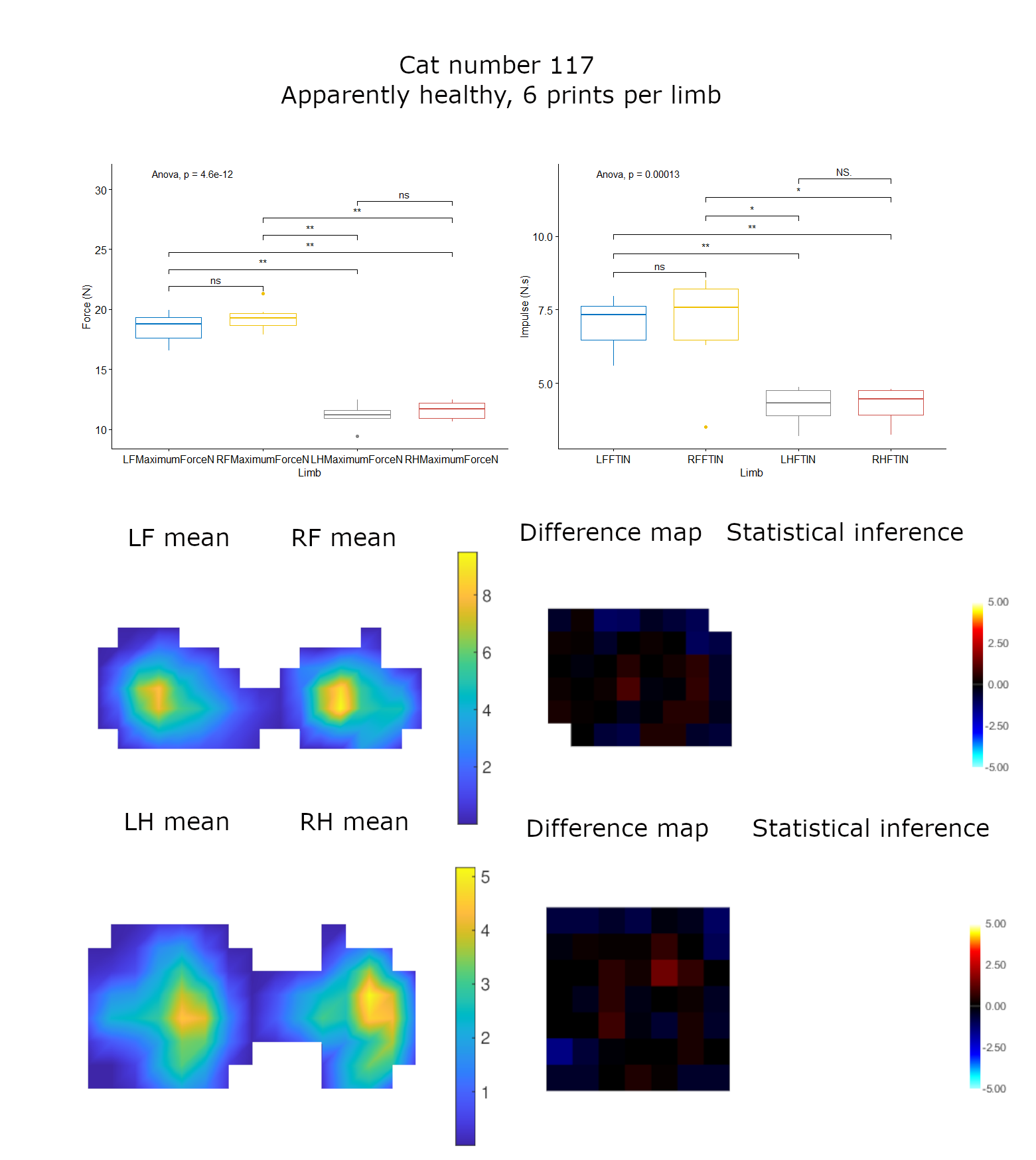

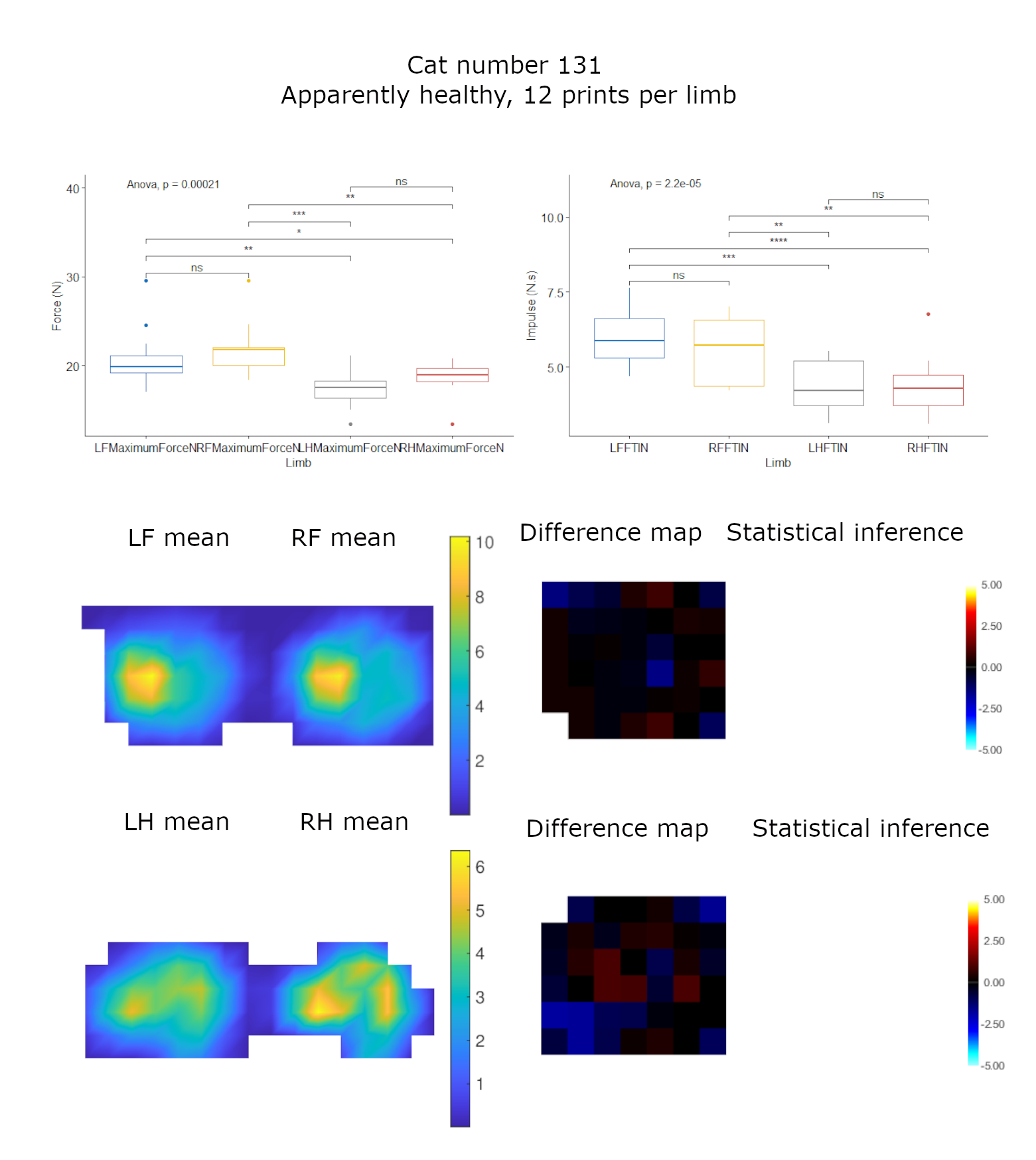

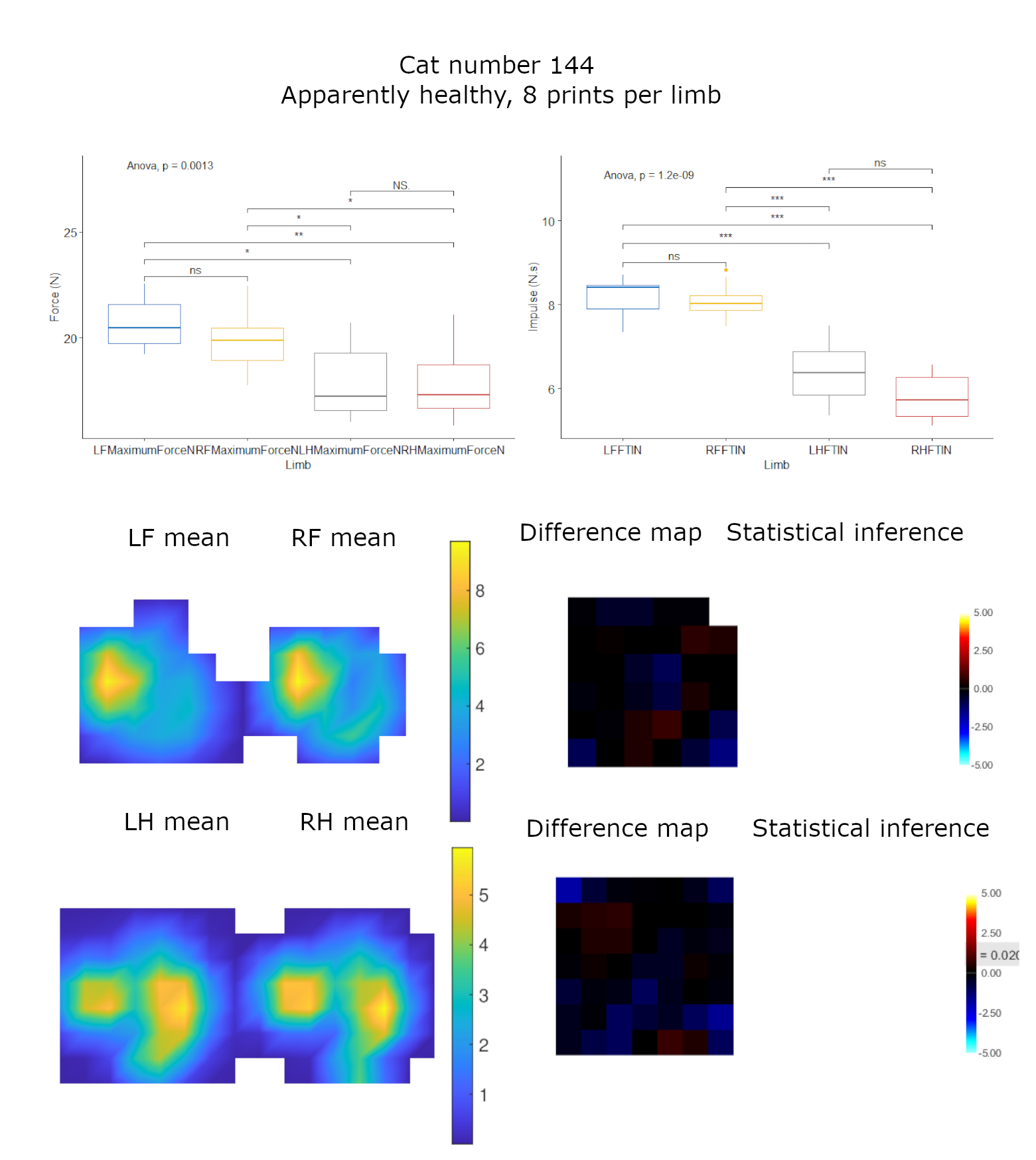

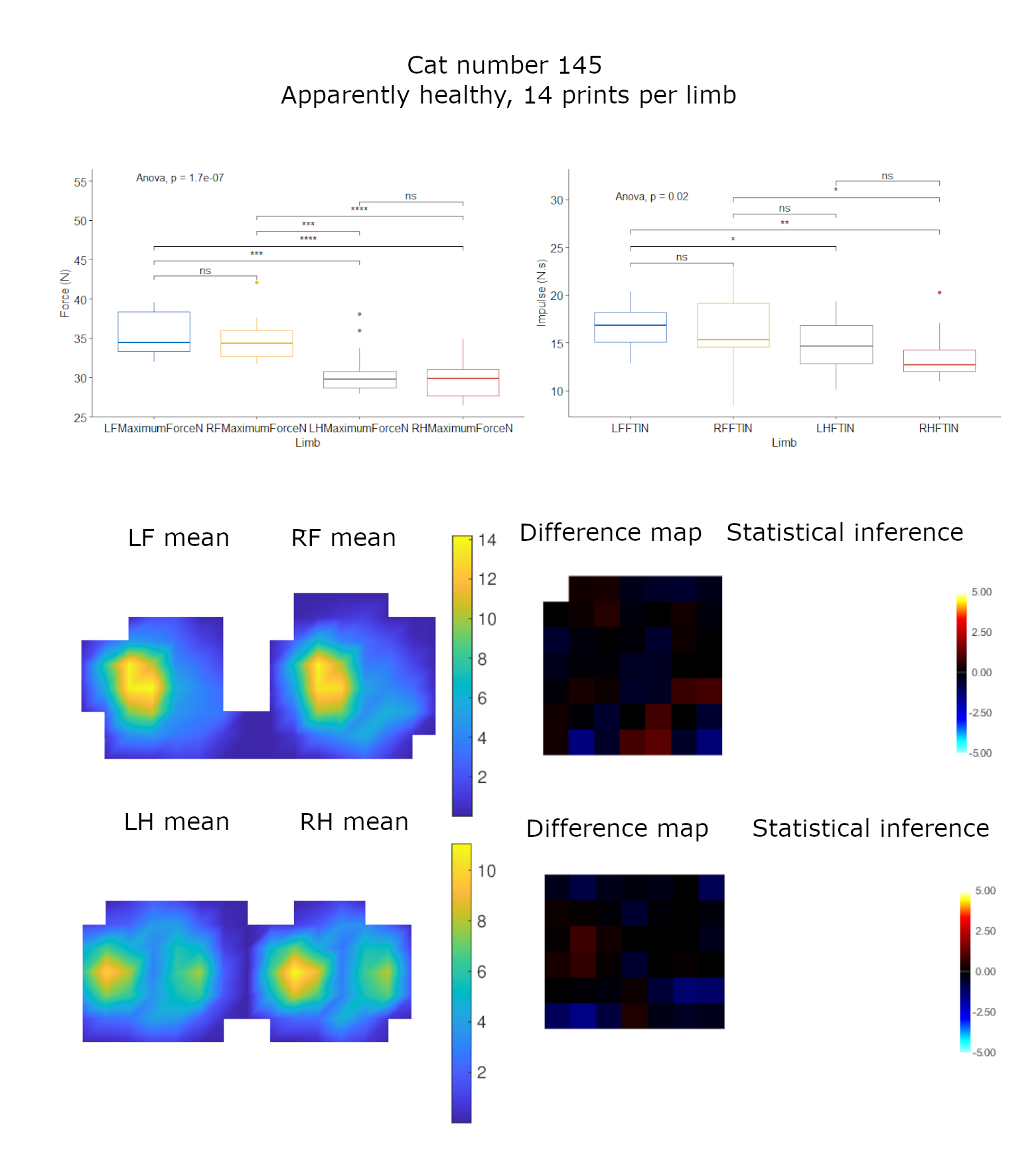

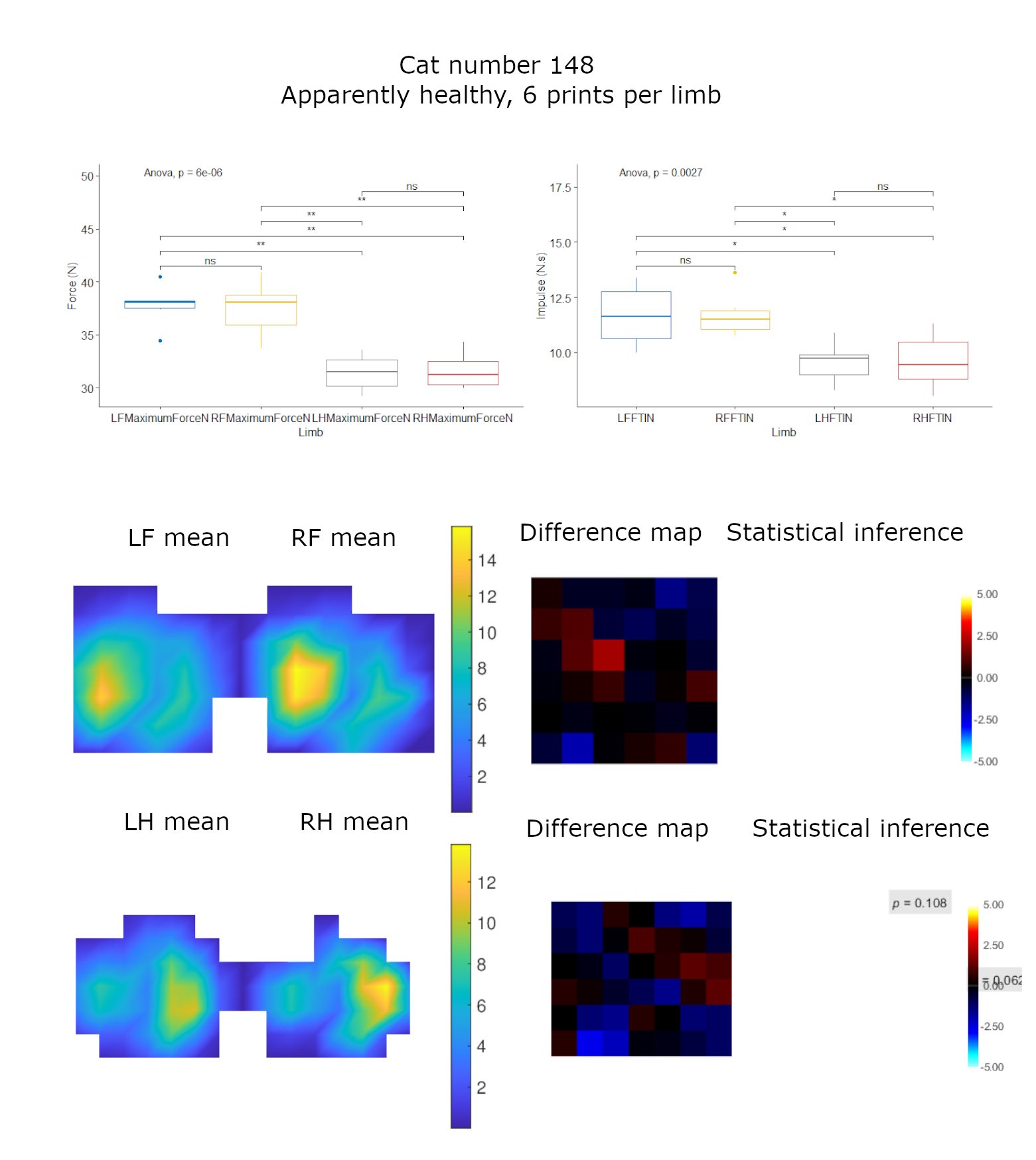

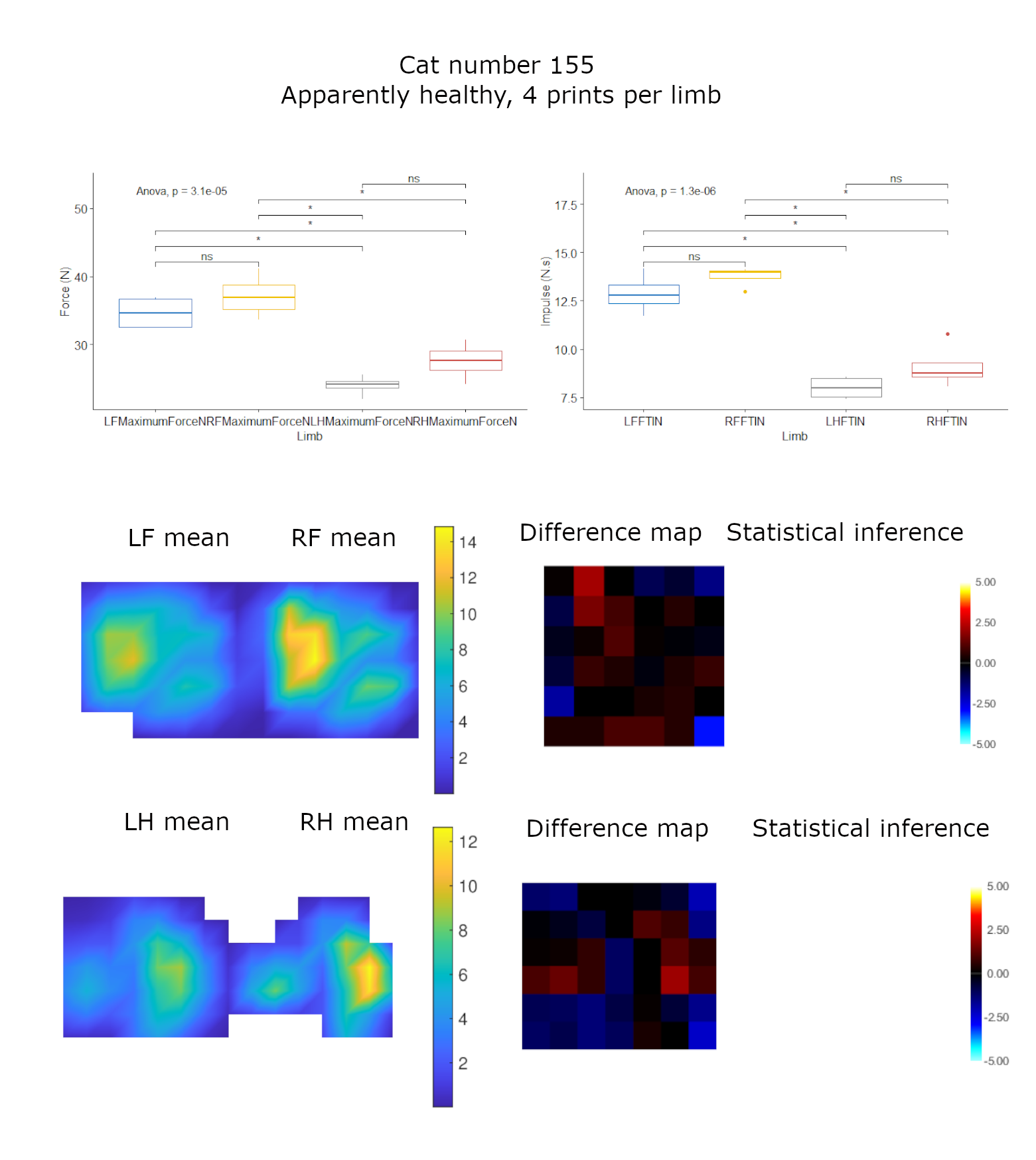

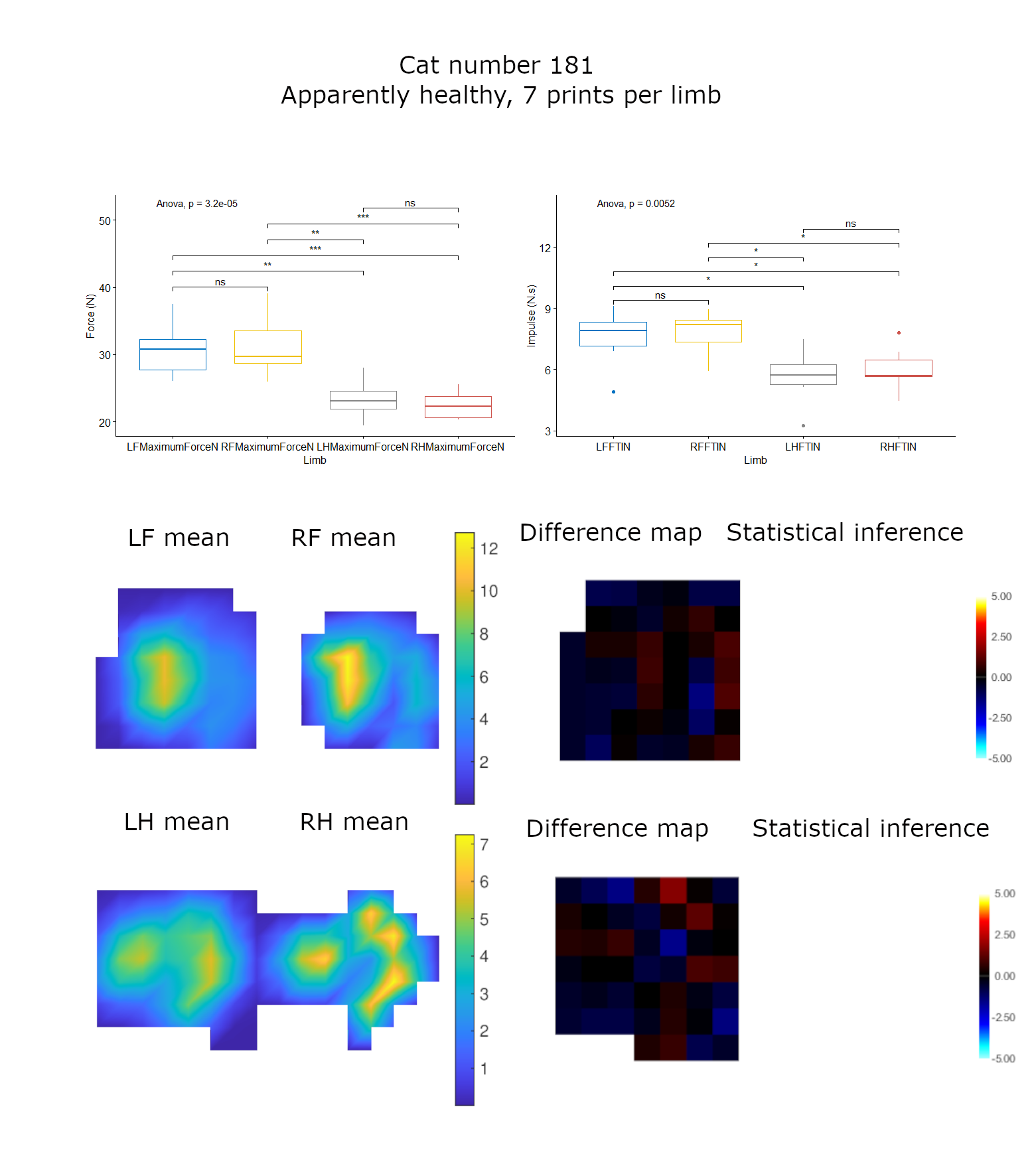

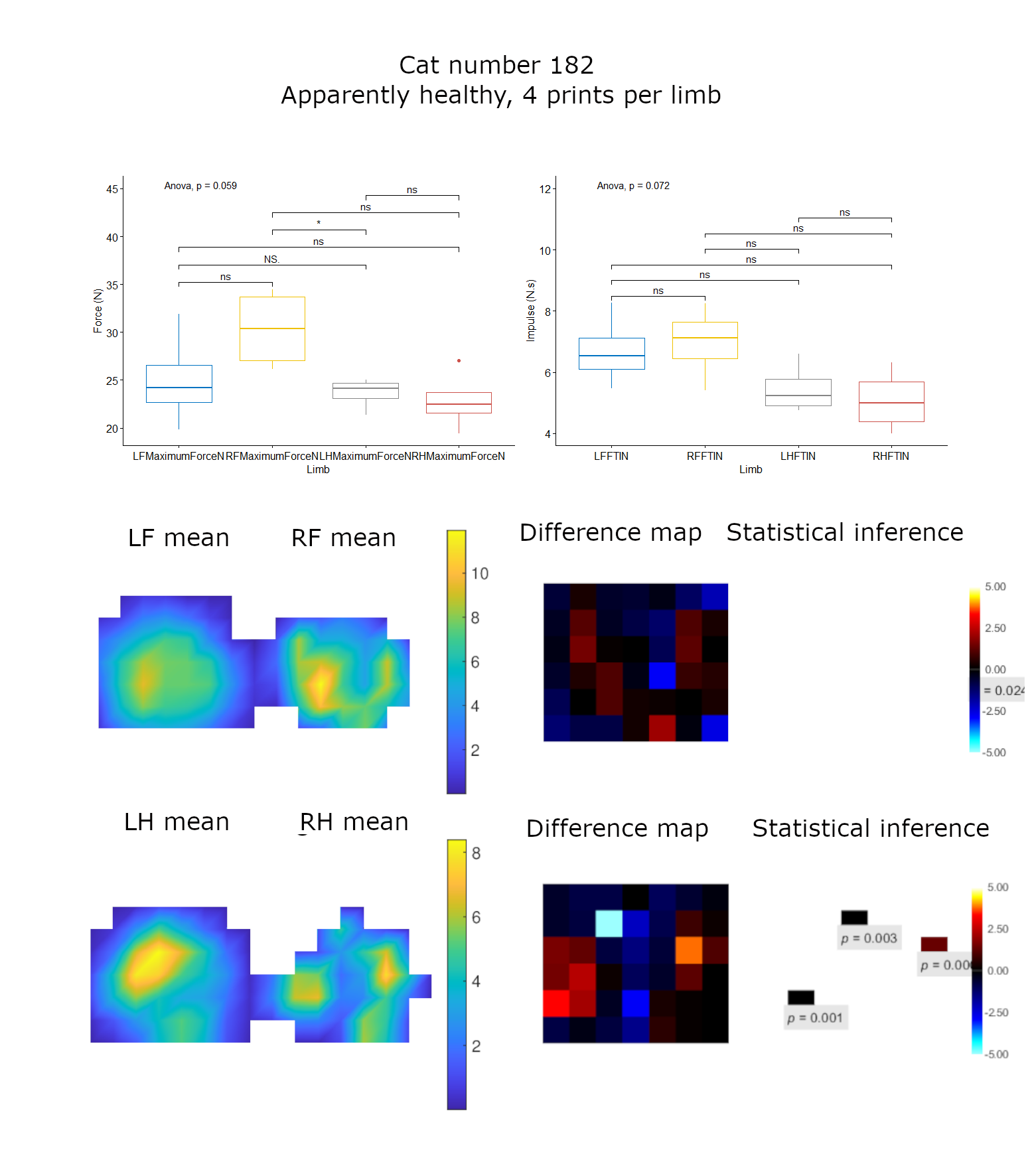

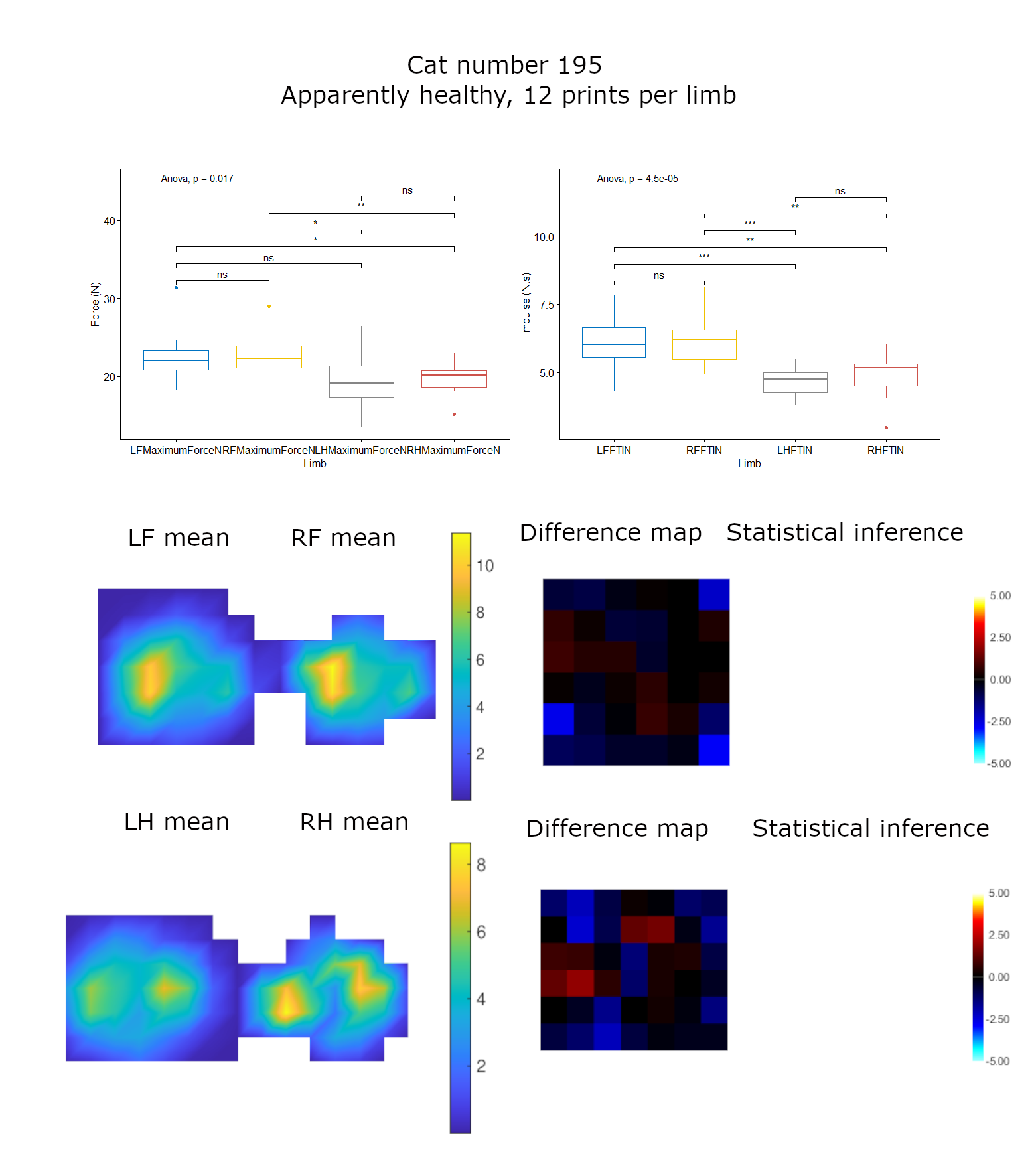

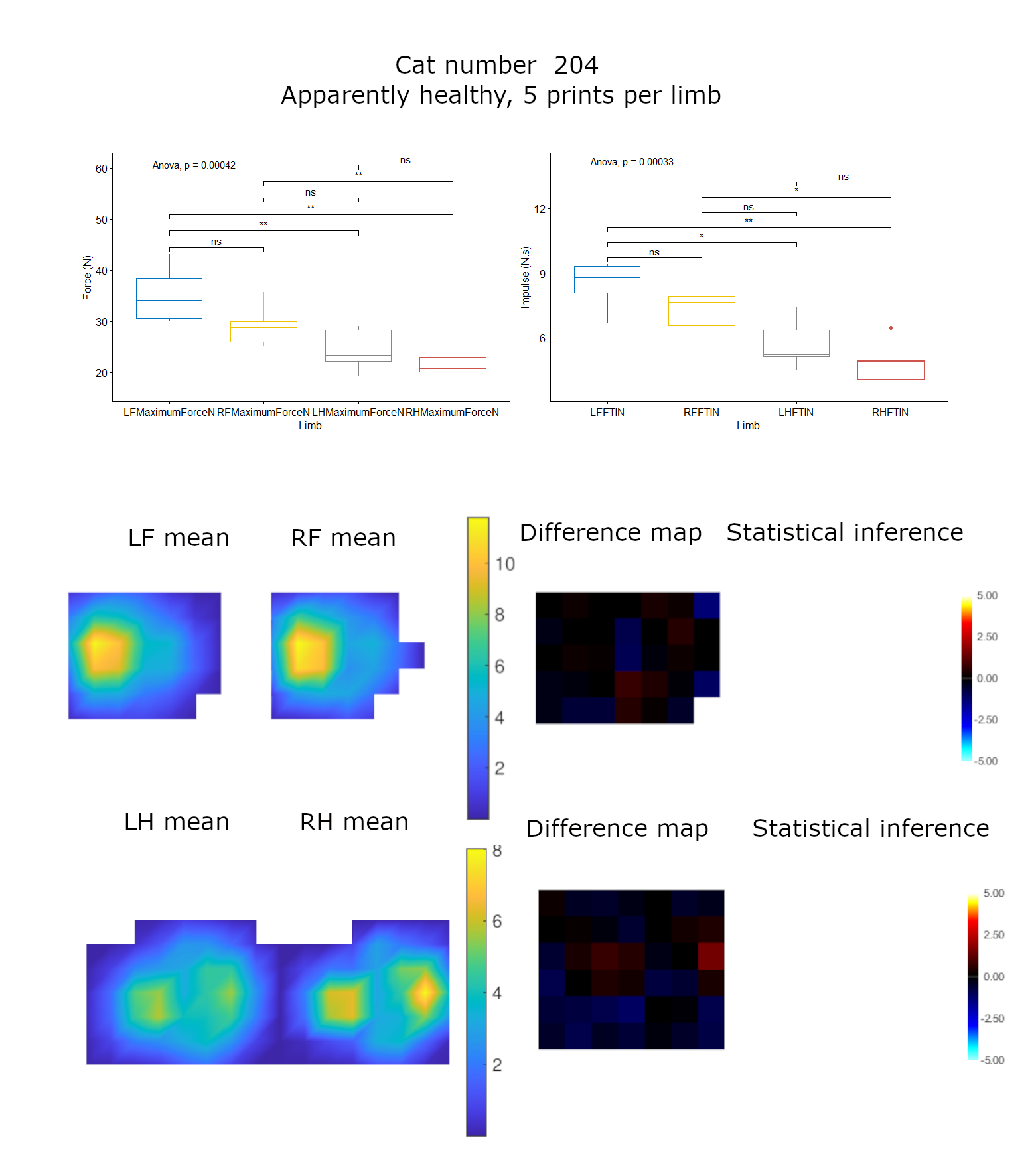

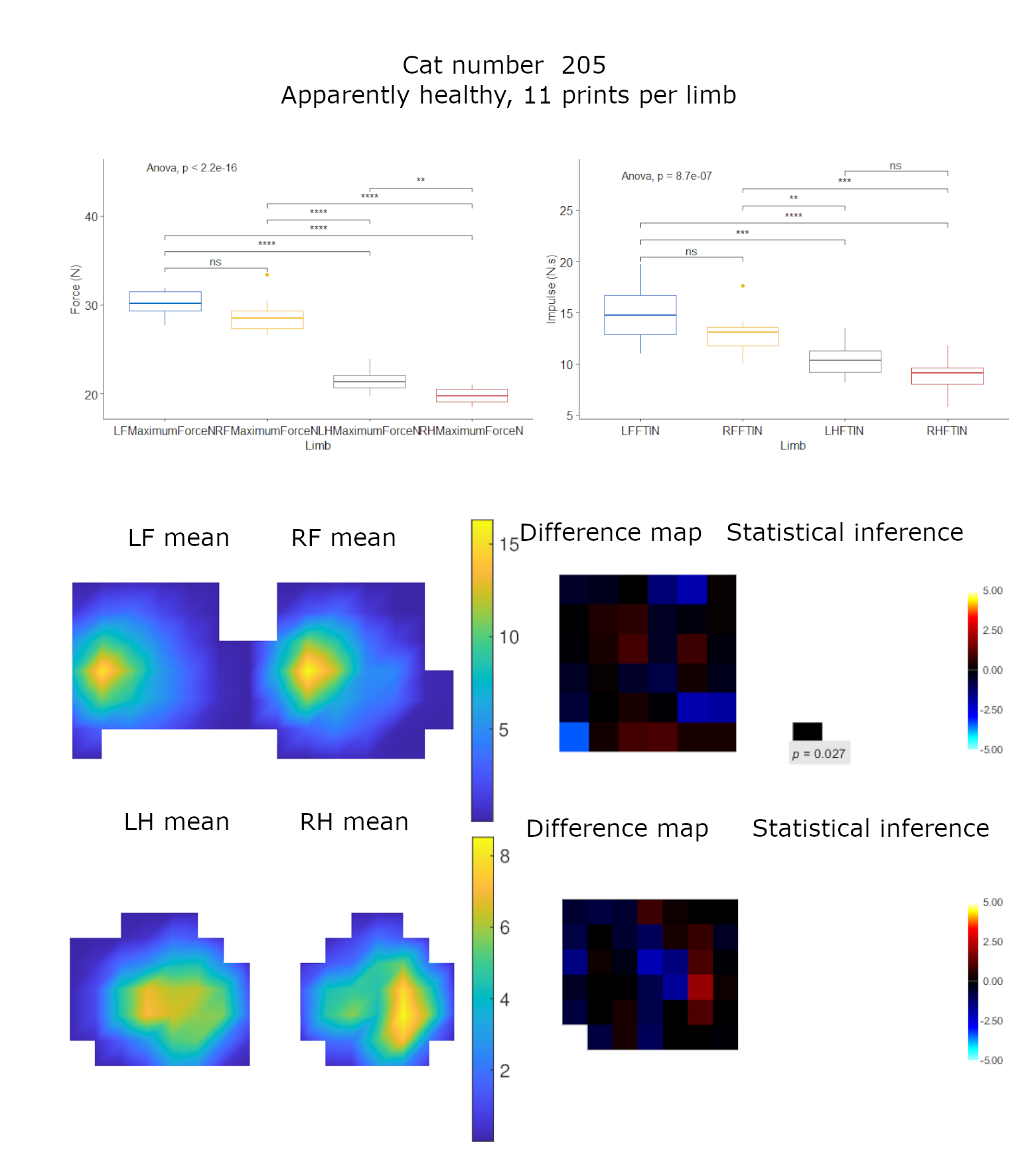


**Musculoskeletal disease**


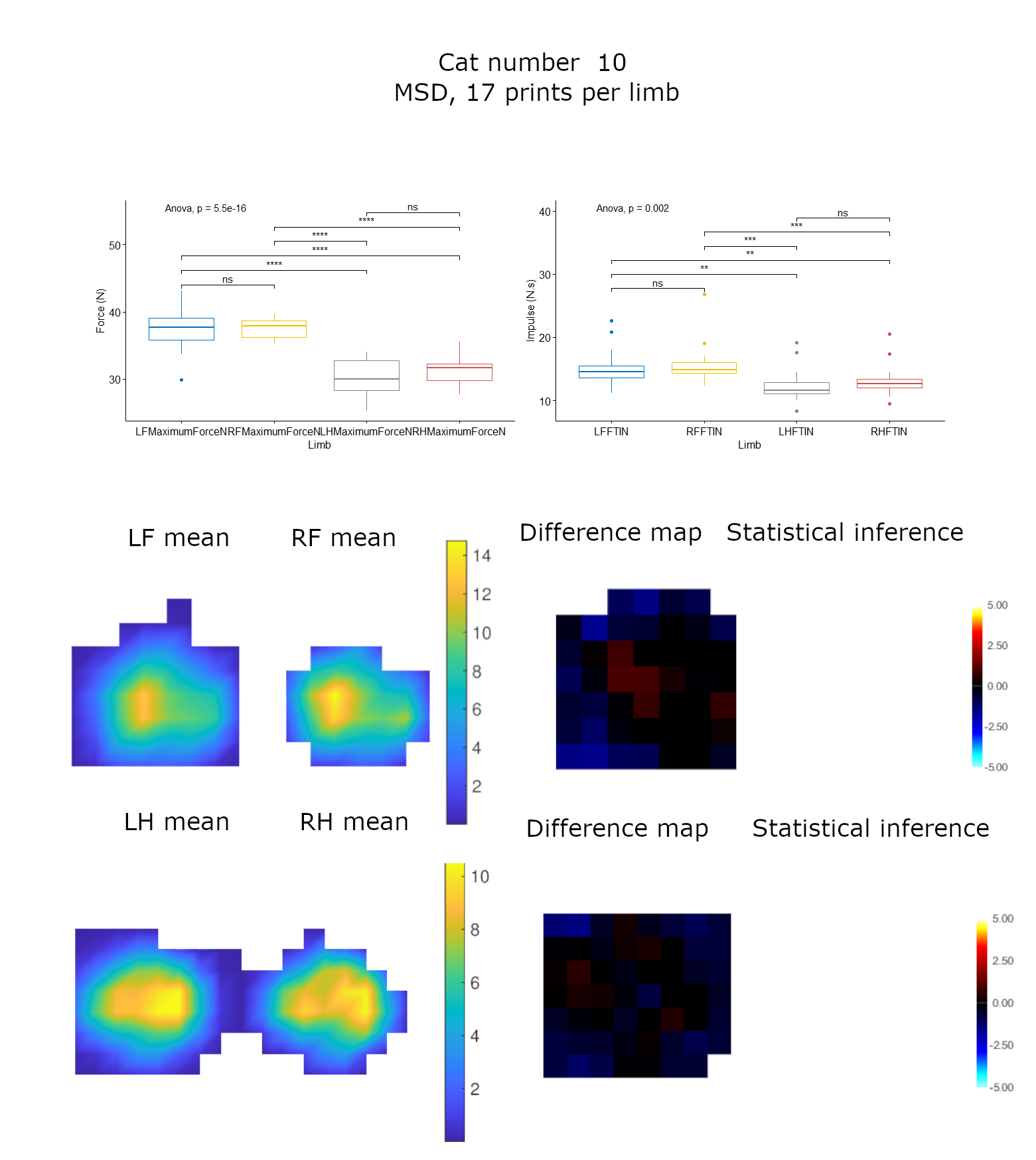

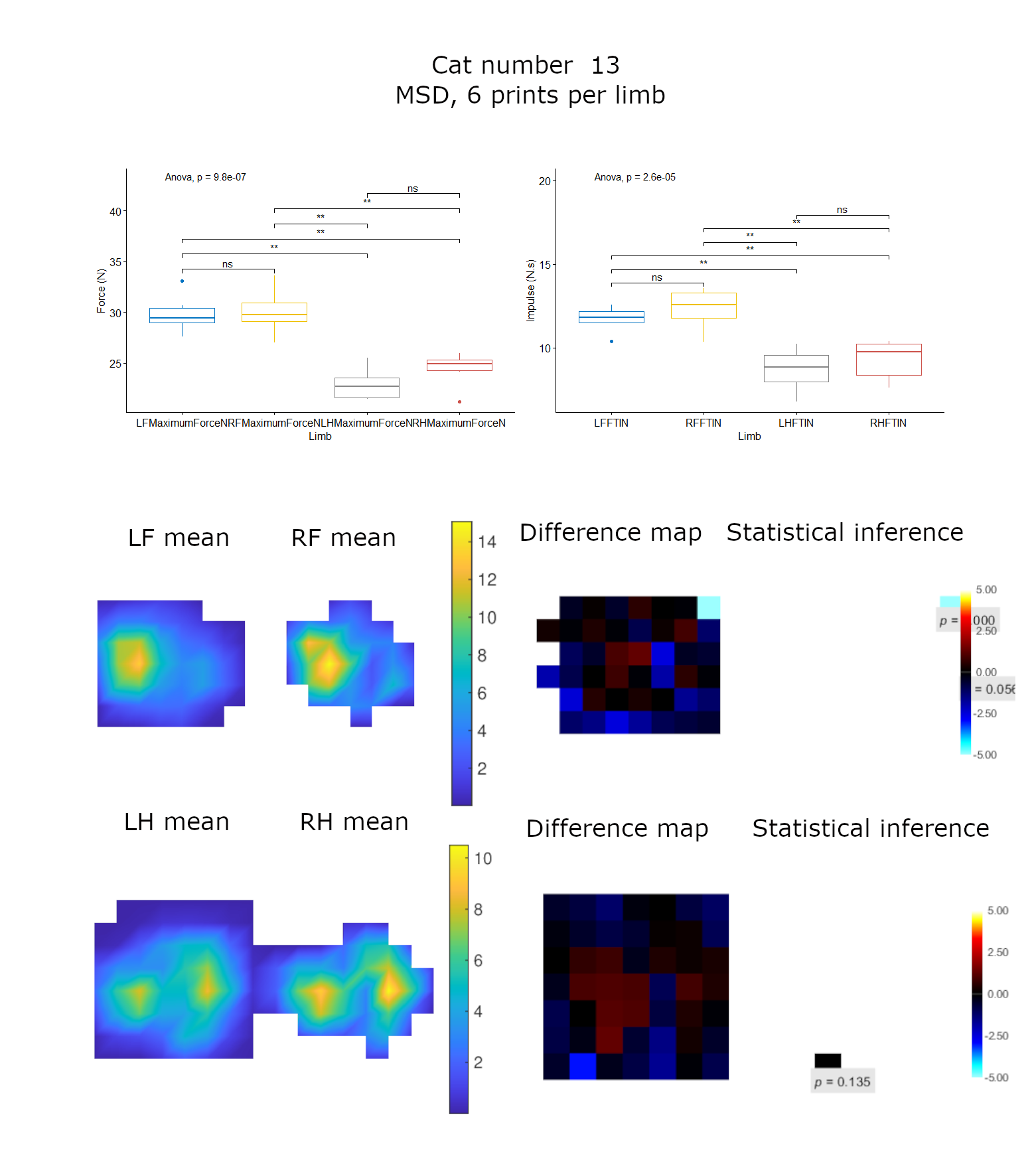

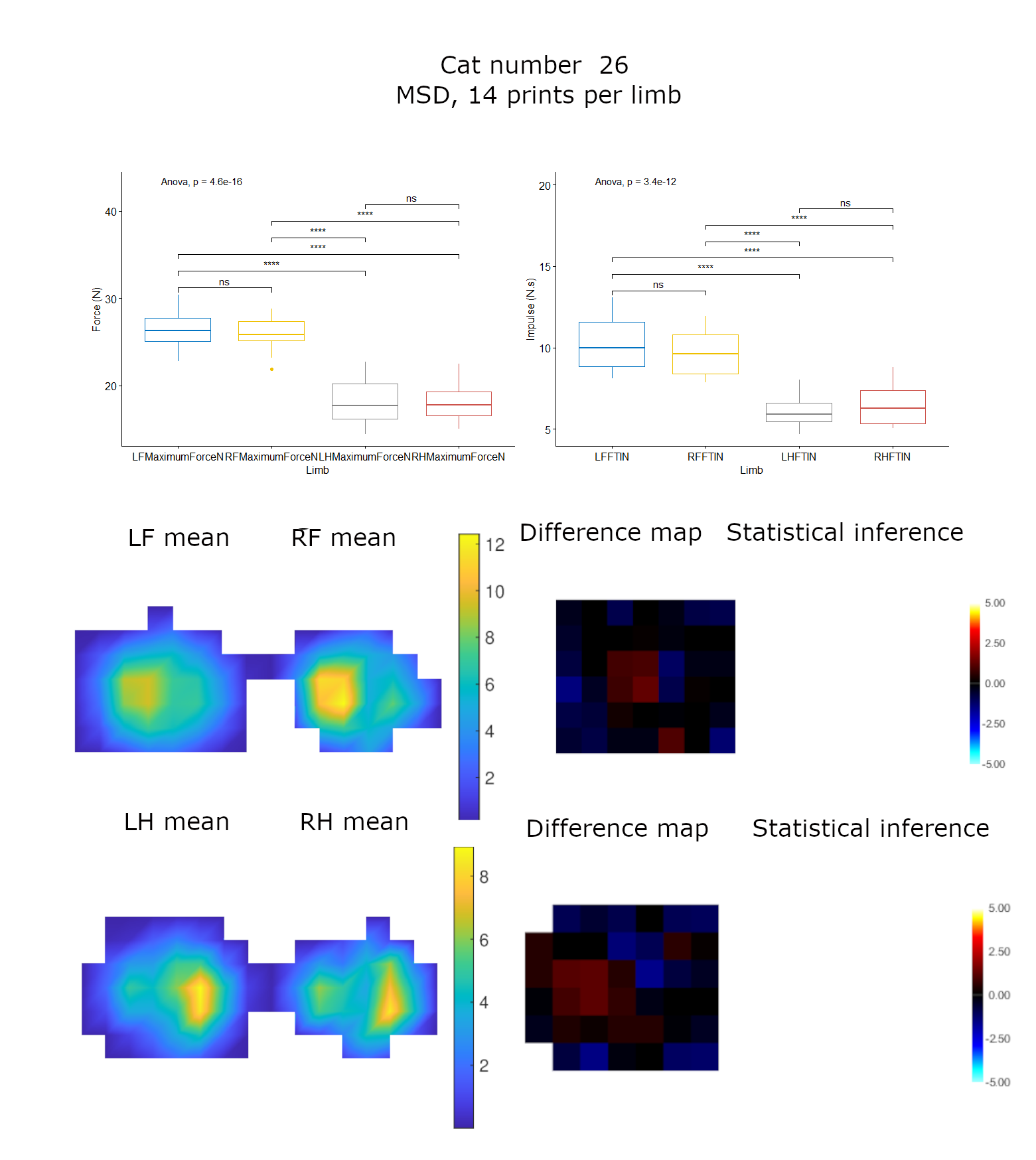

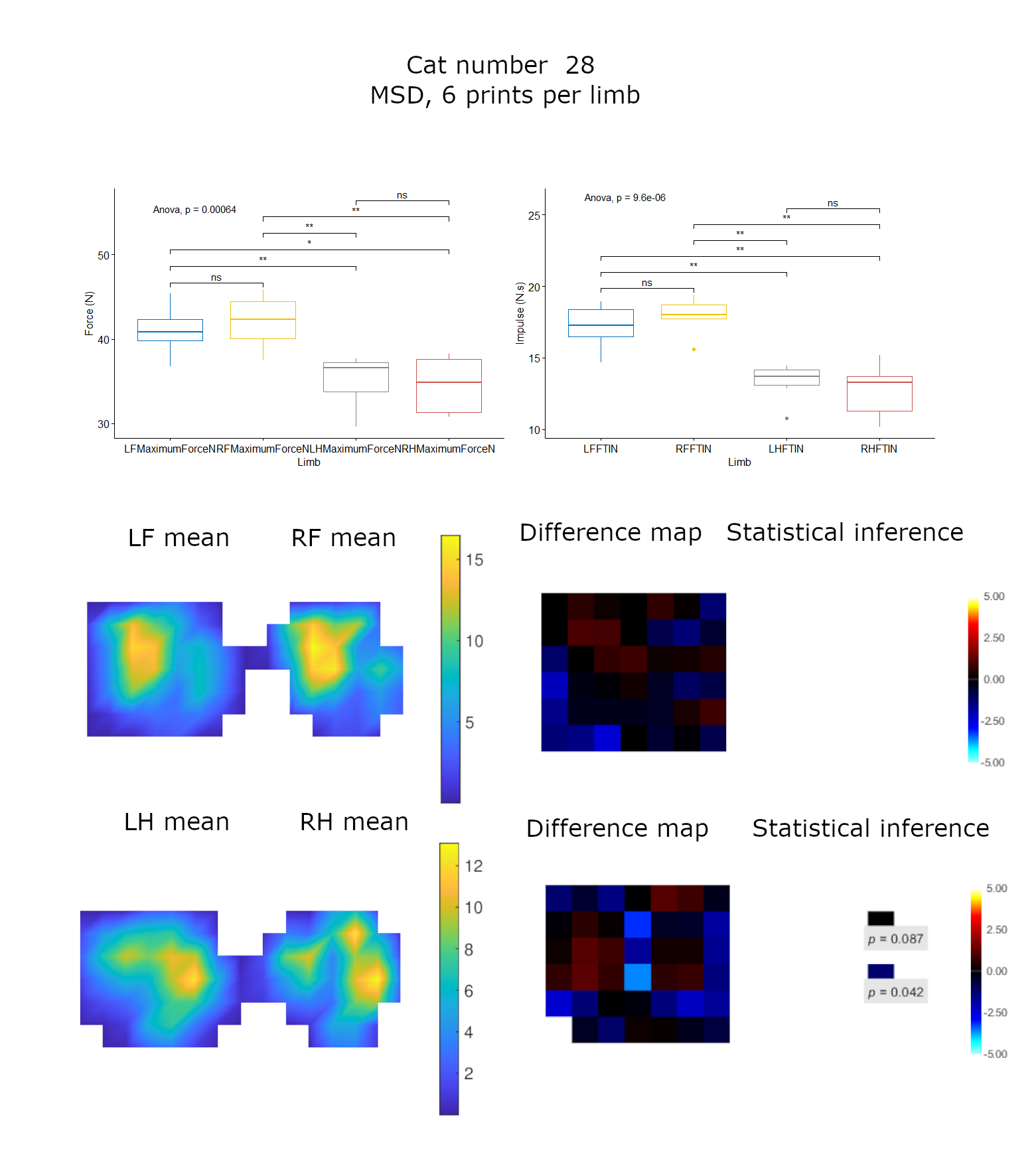

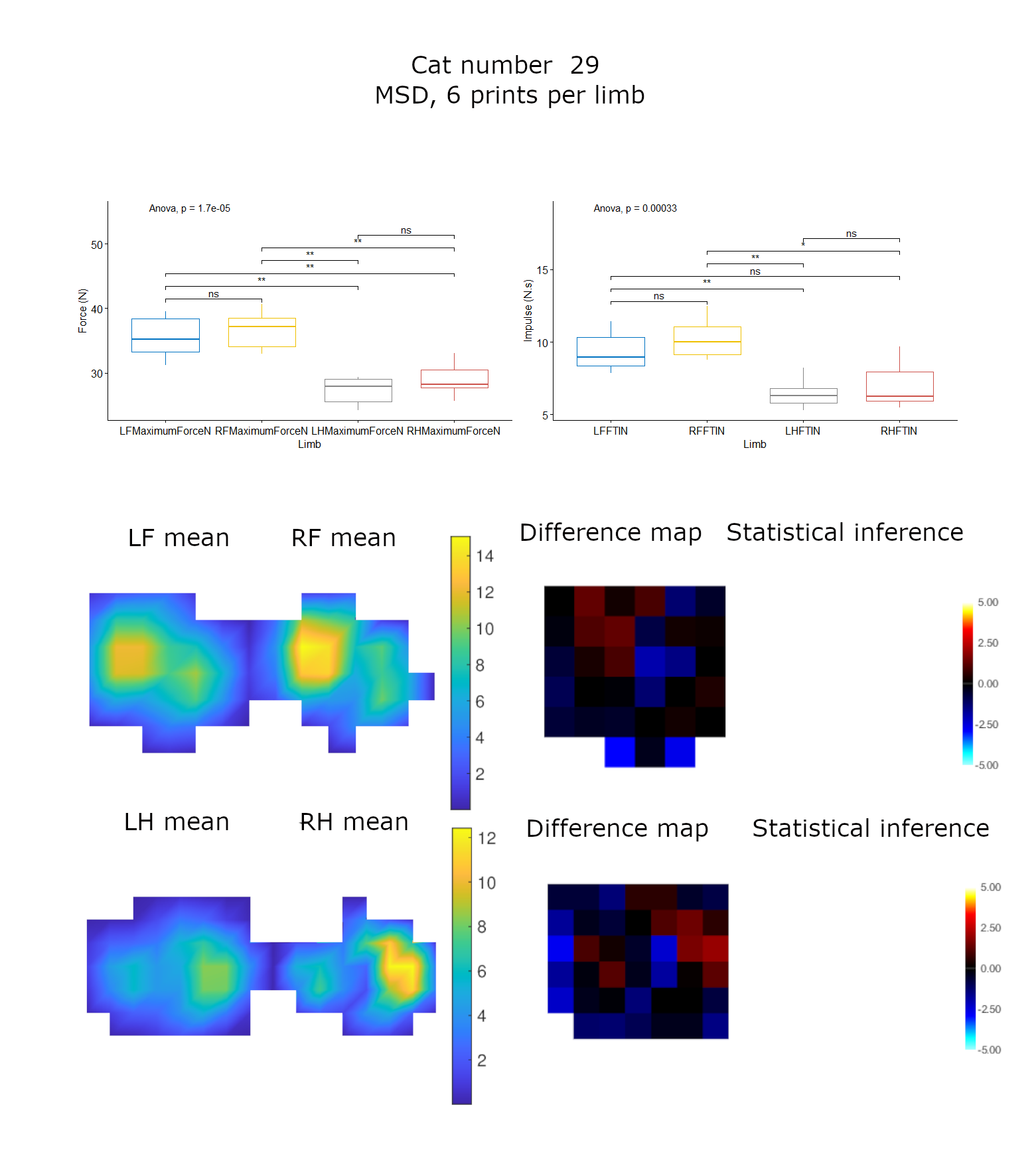

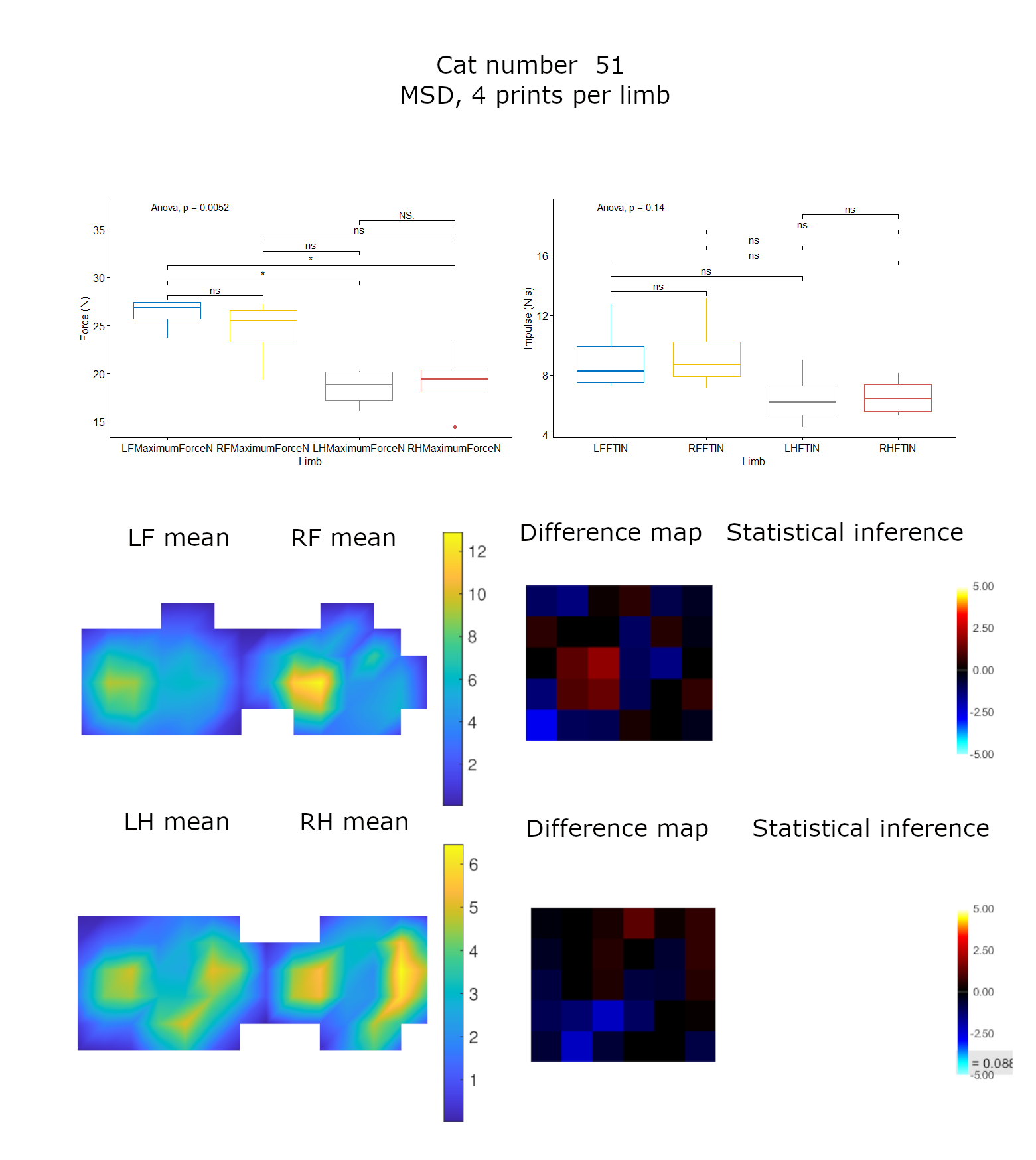

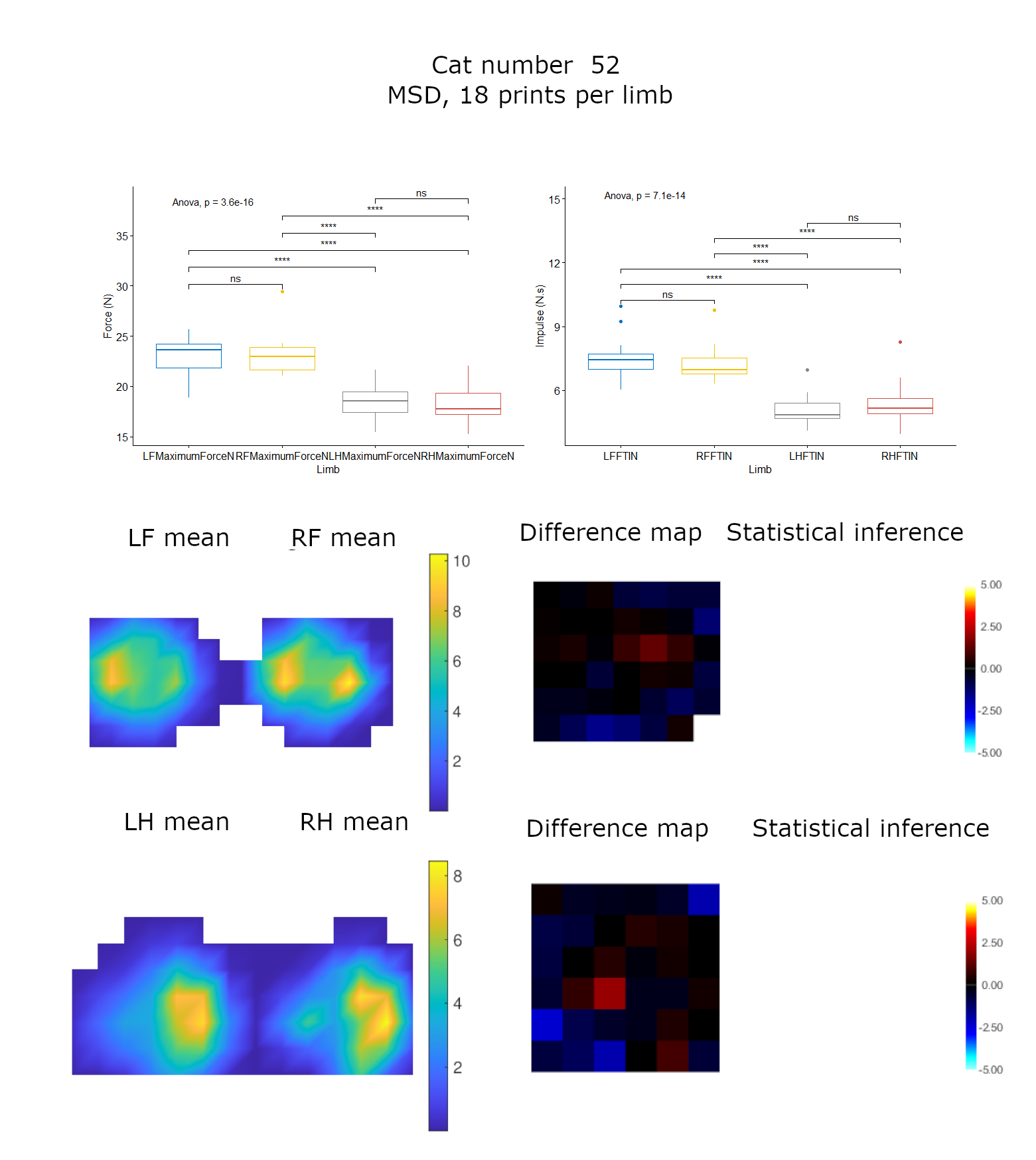

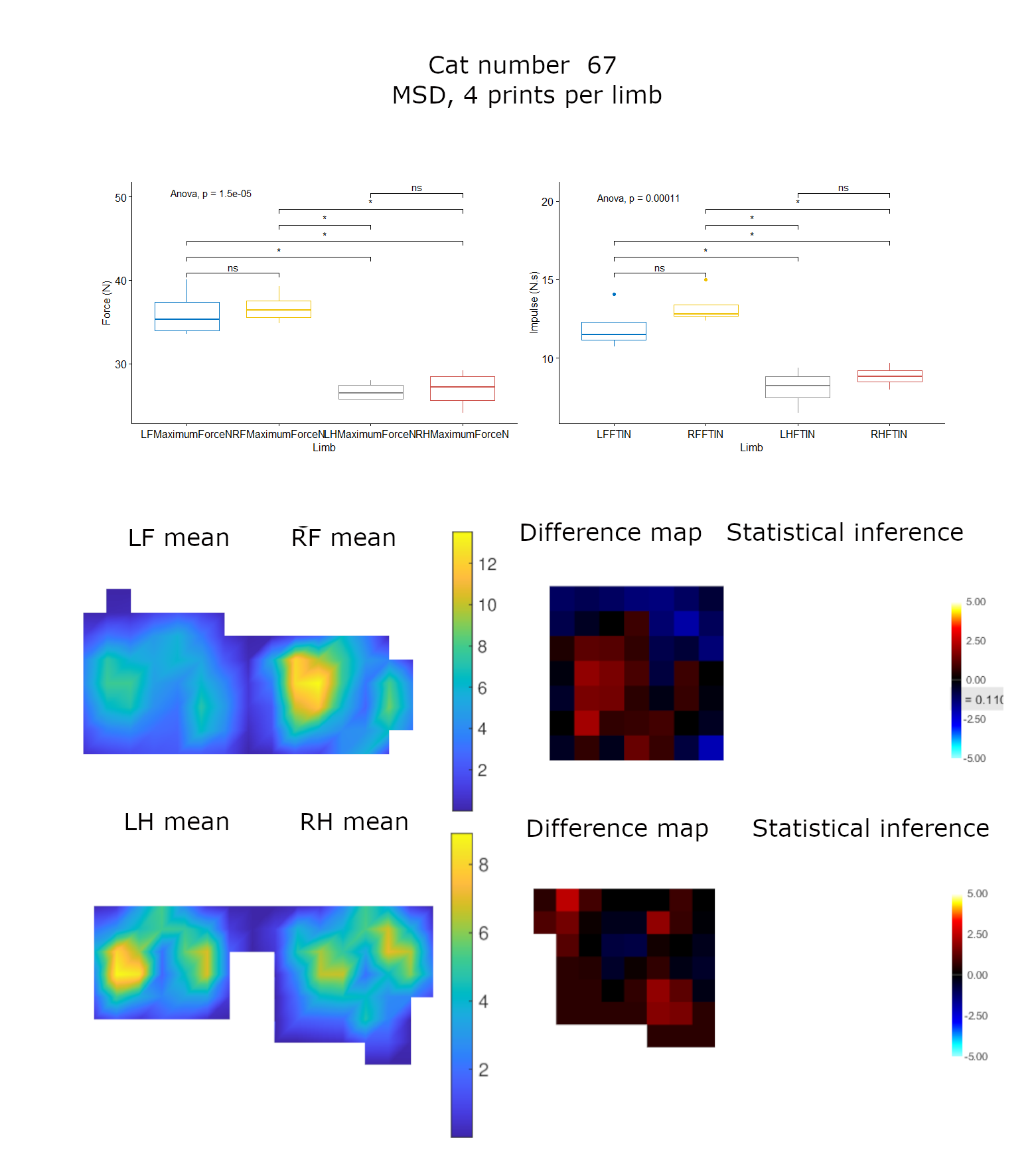

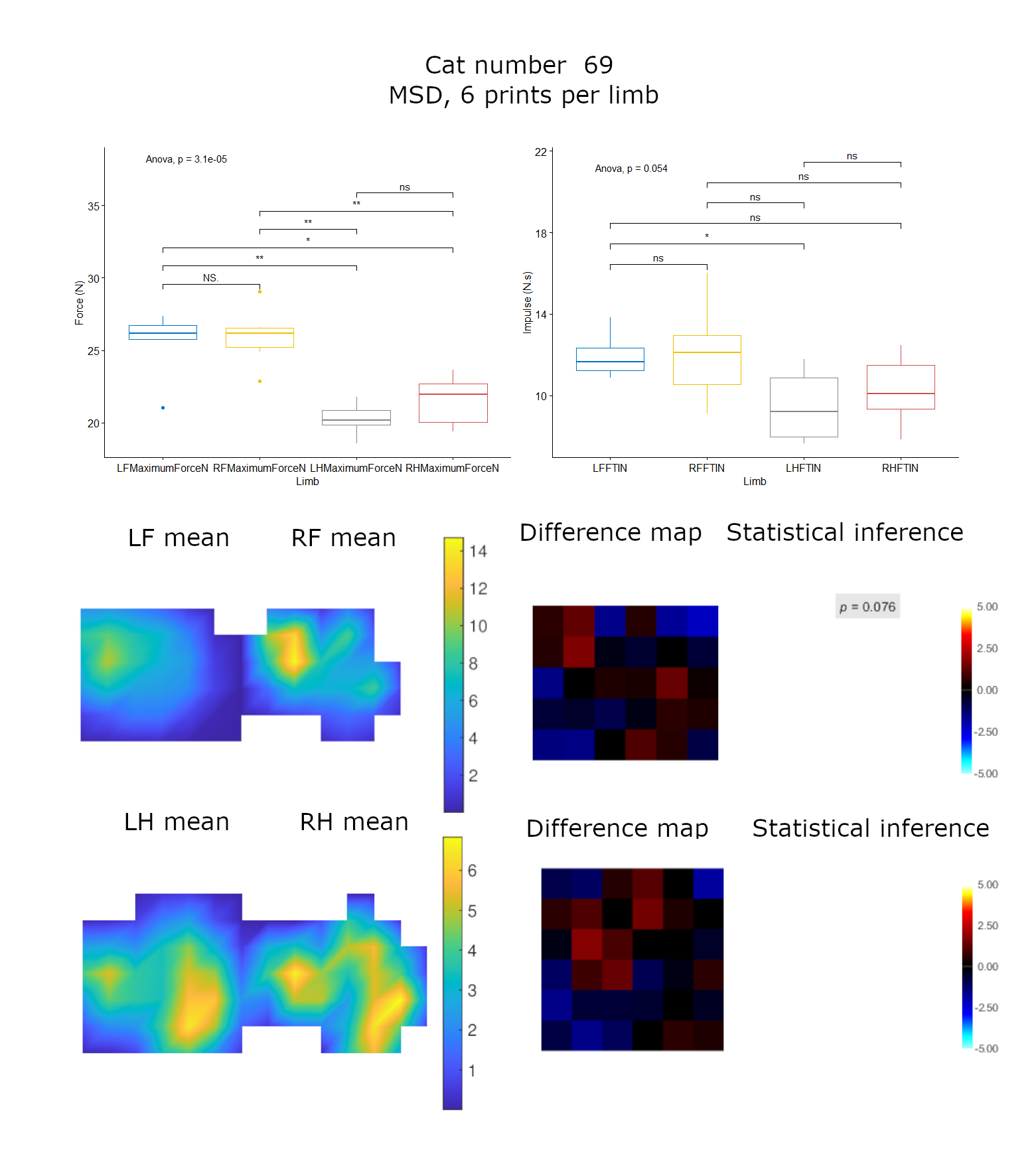

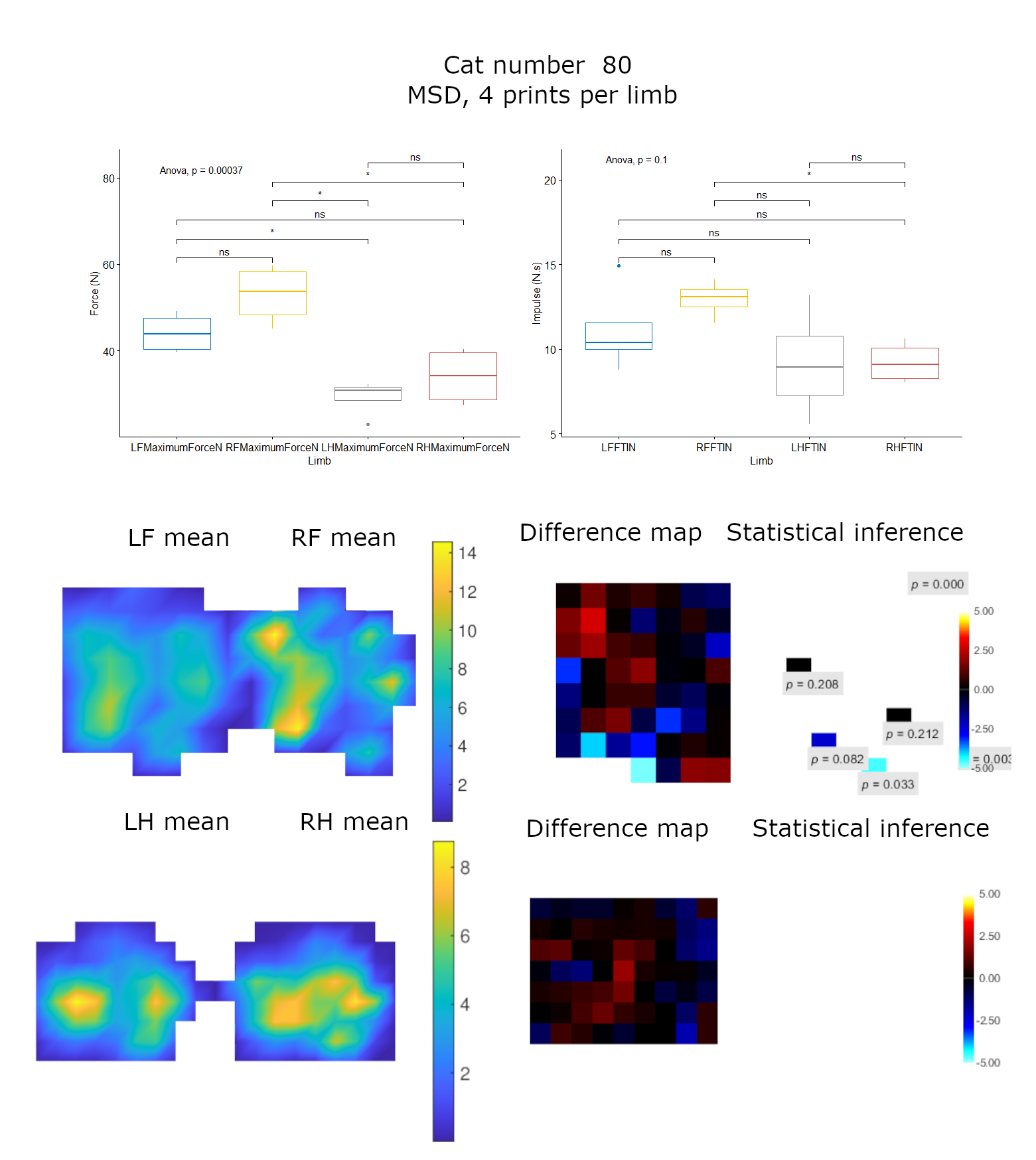

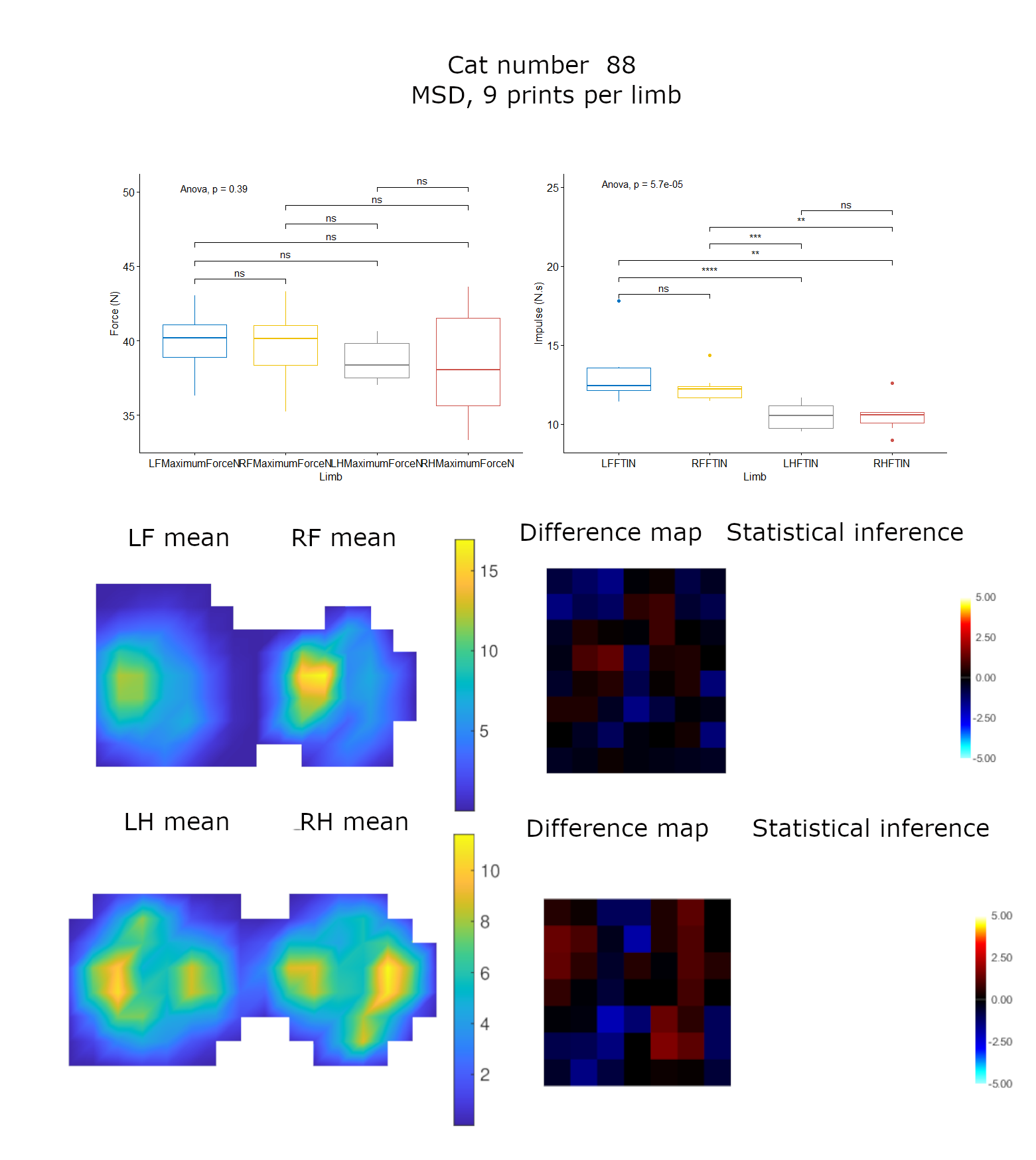

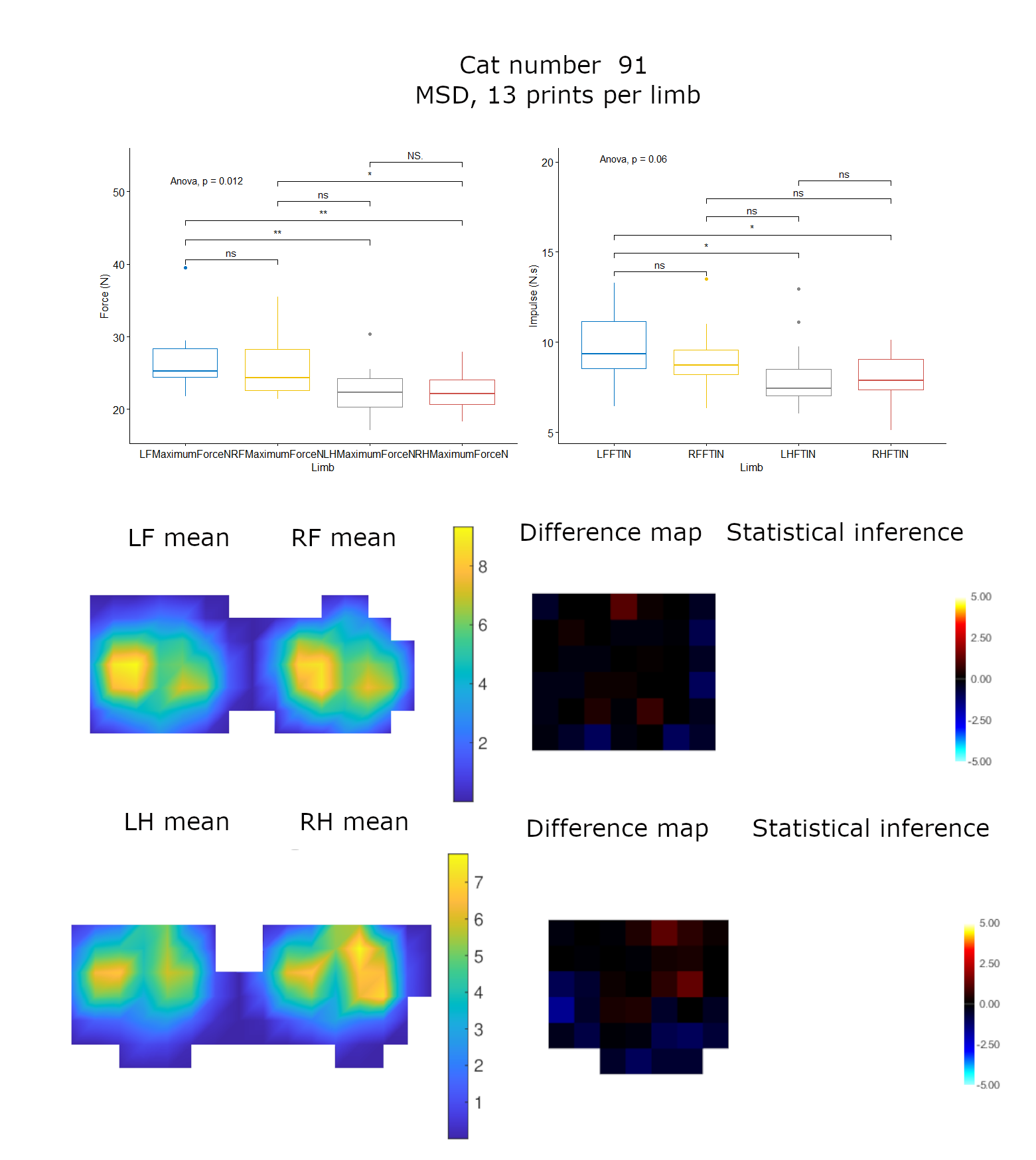

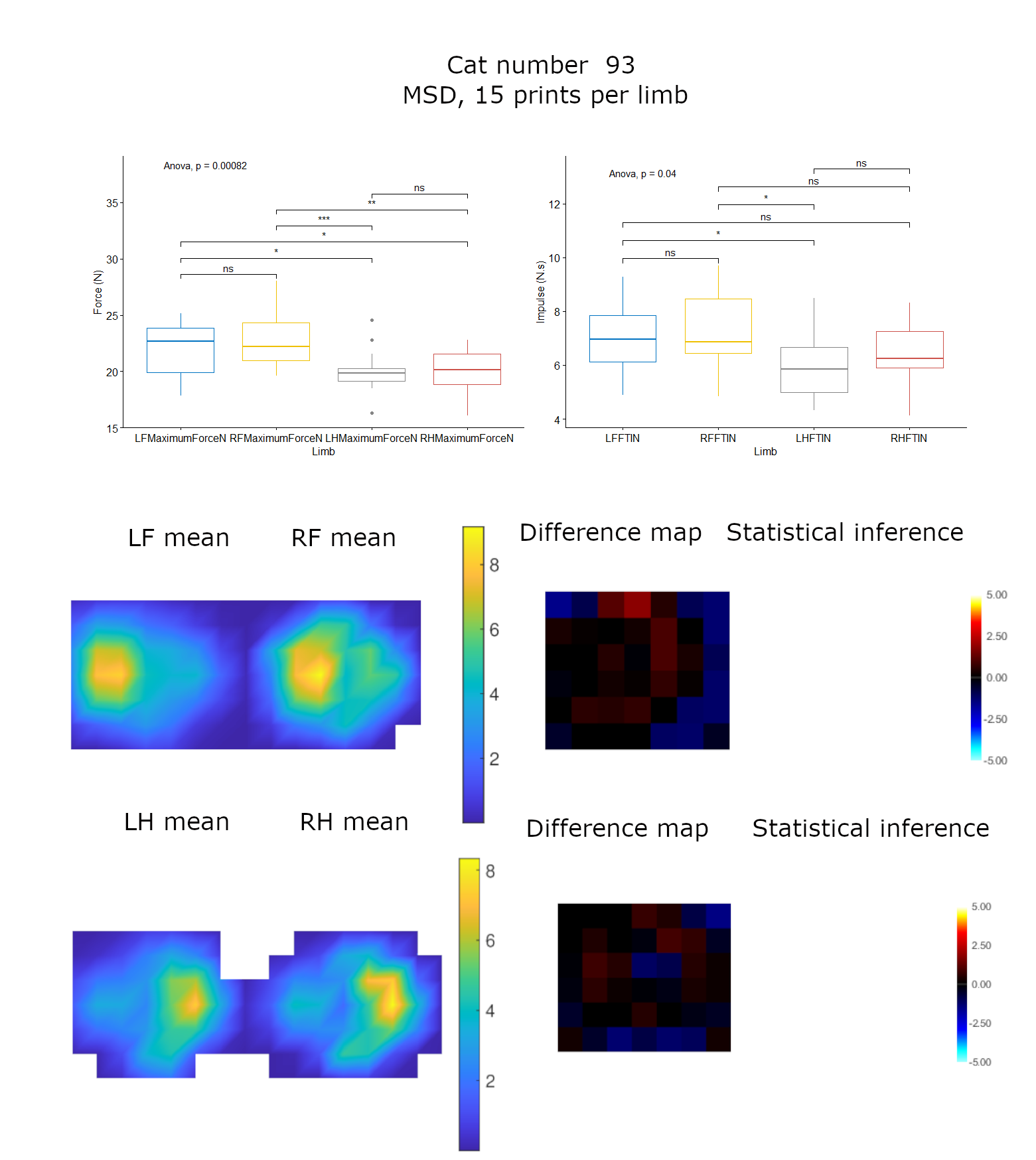

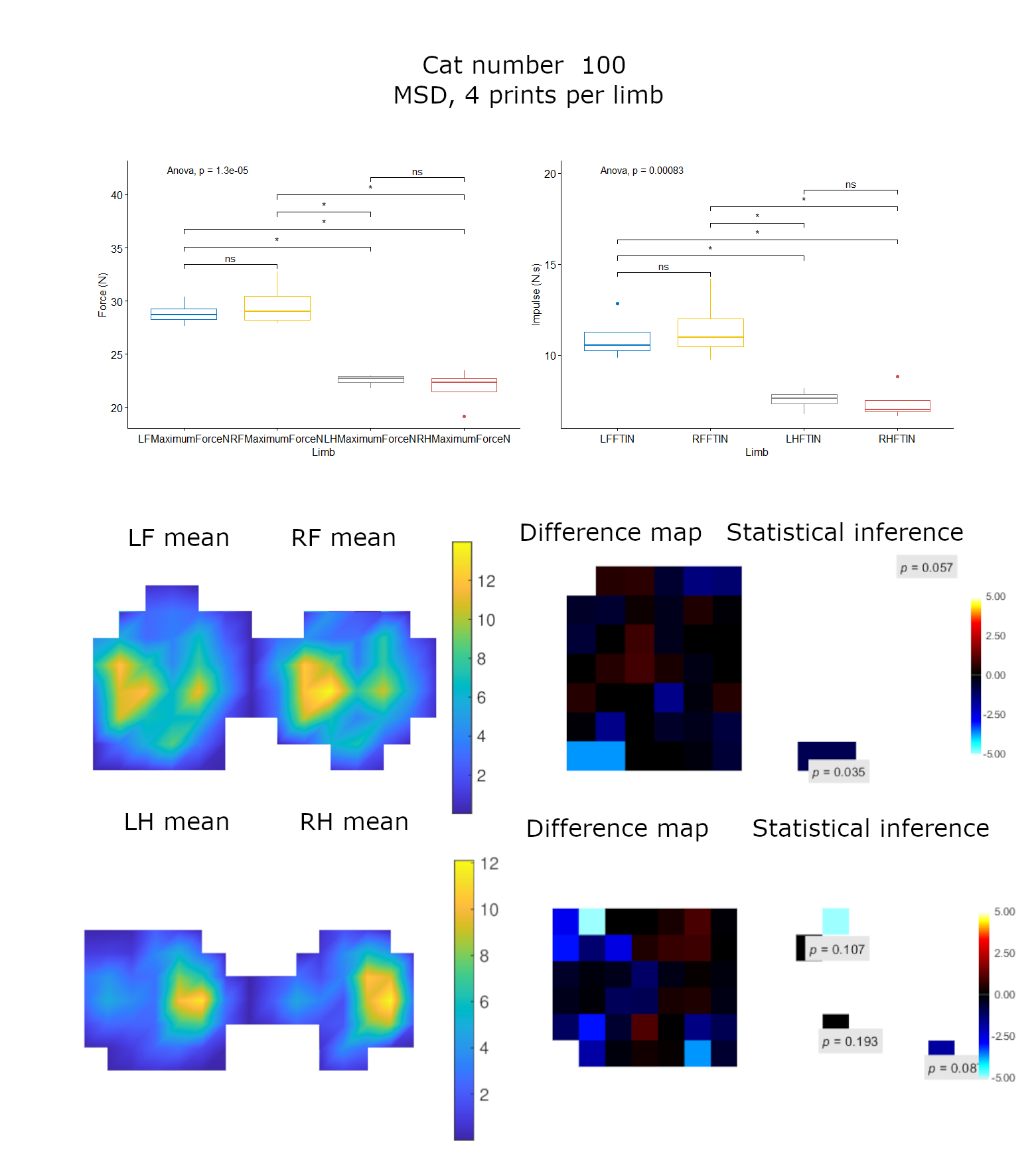

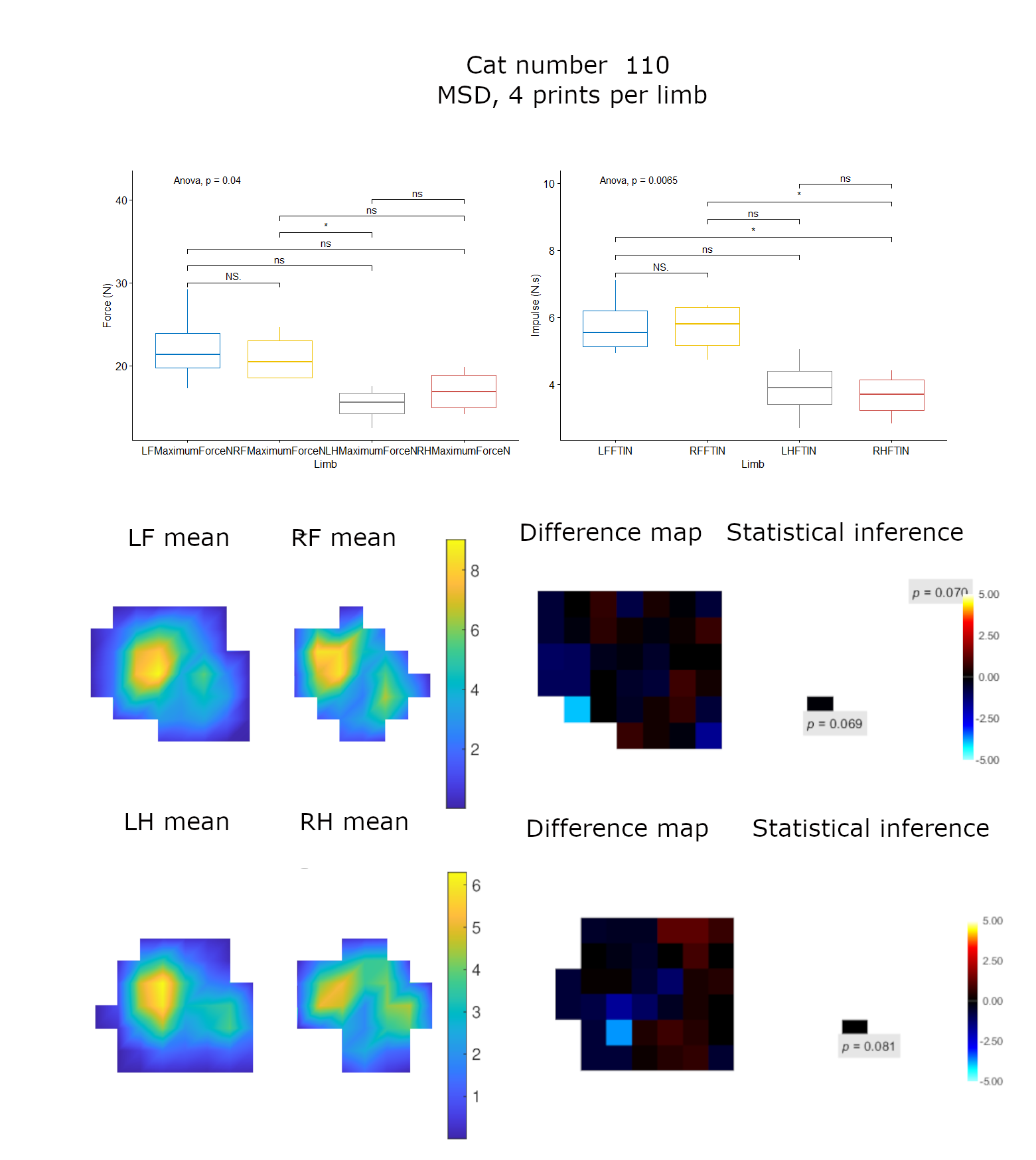

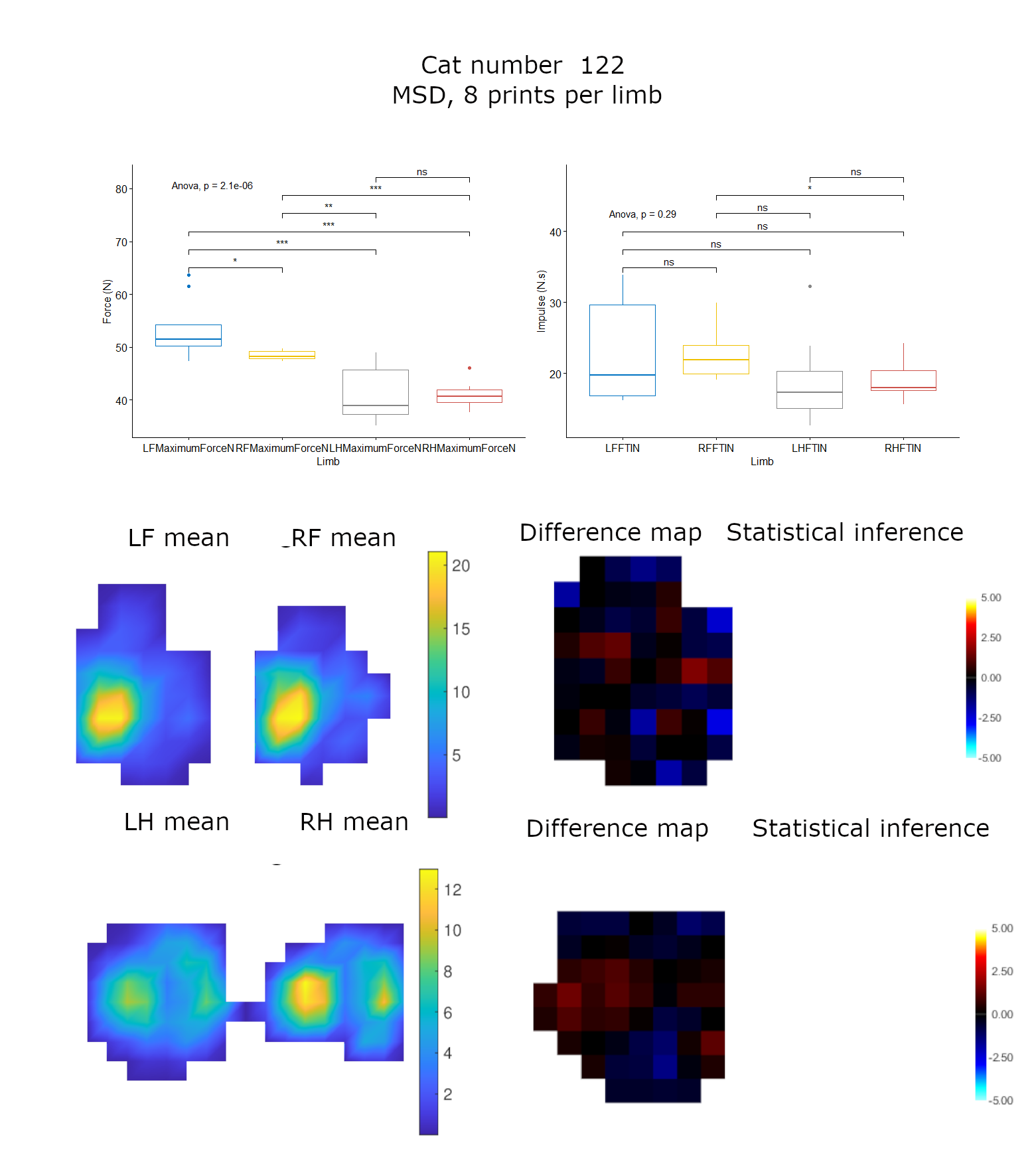

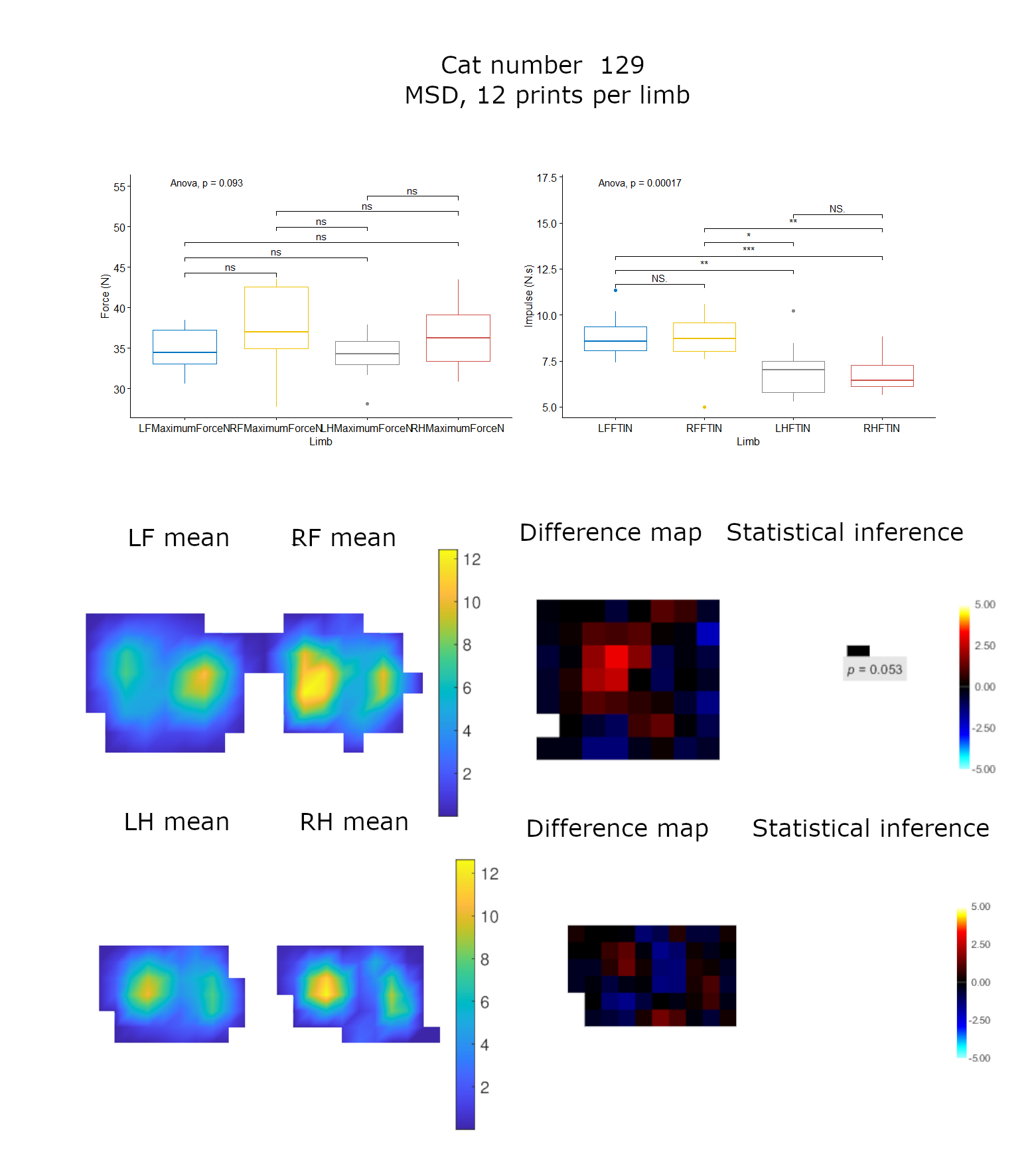

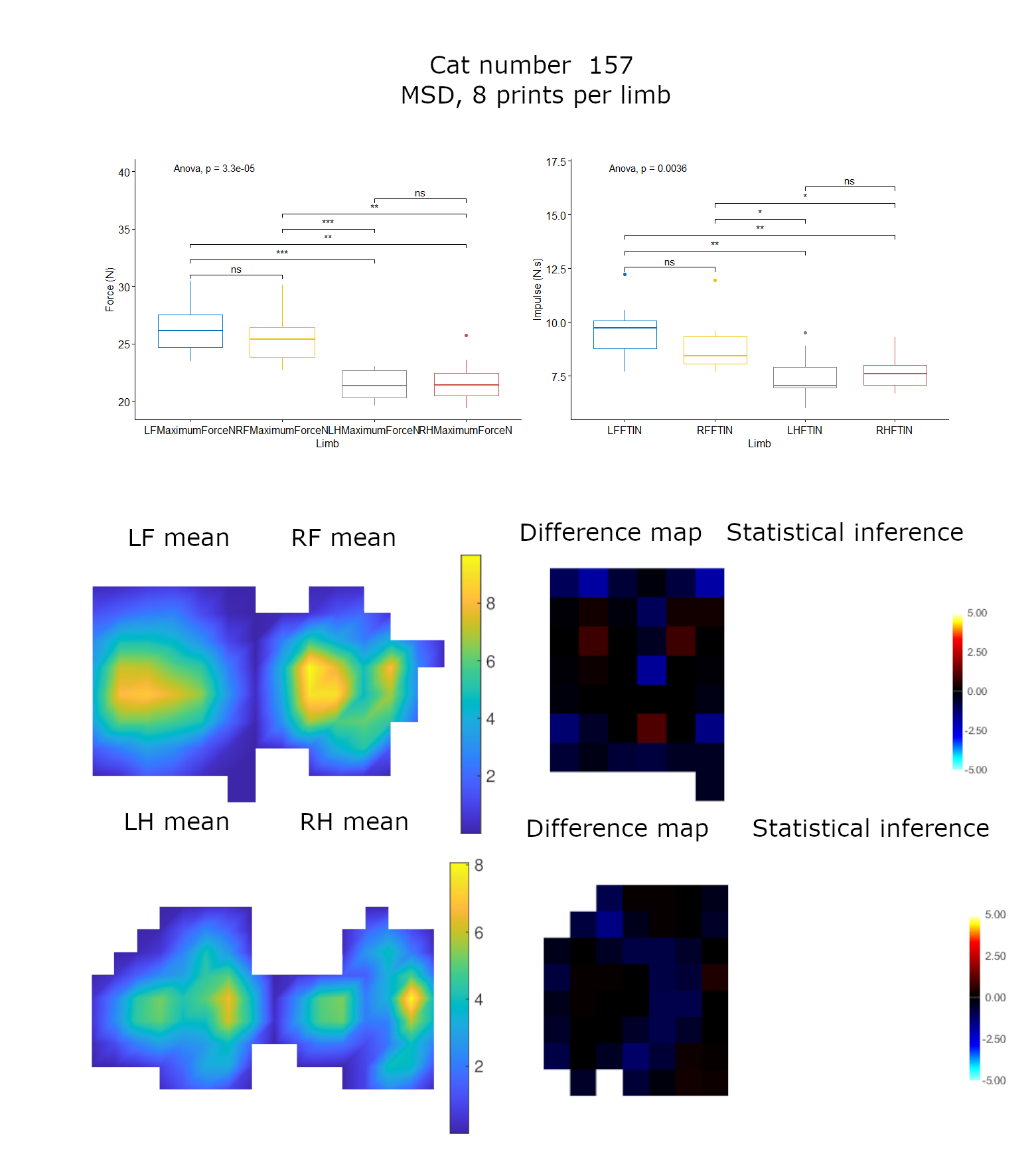

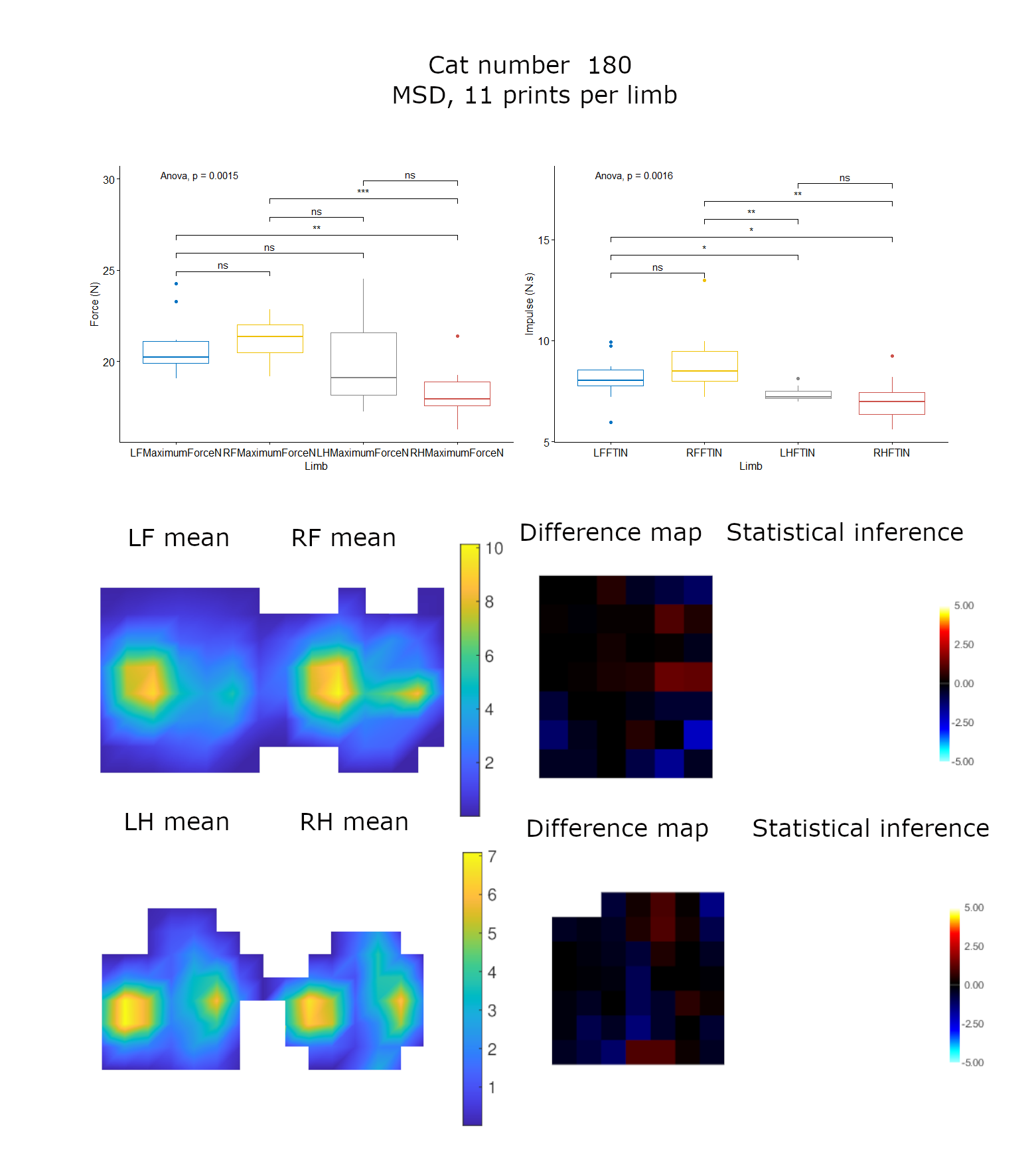

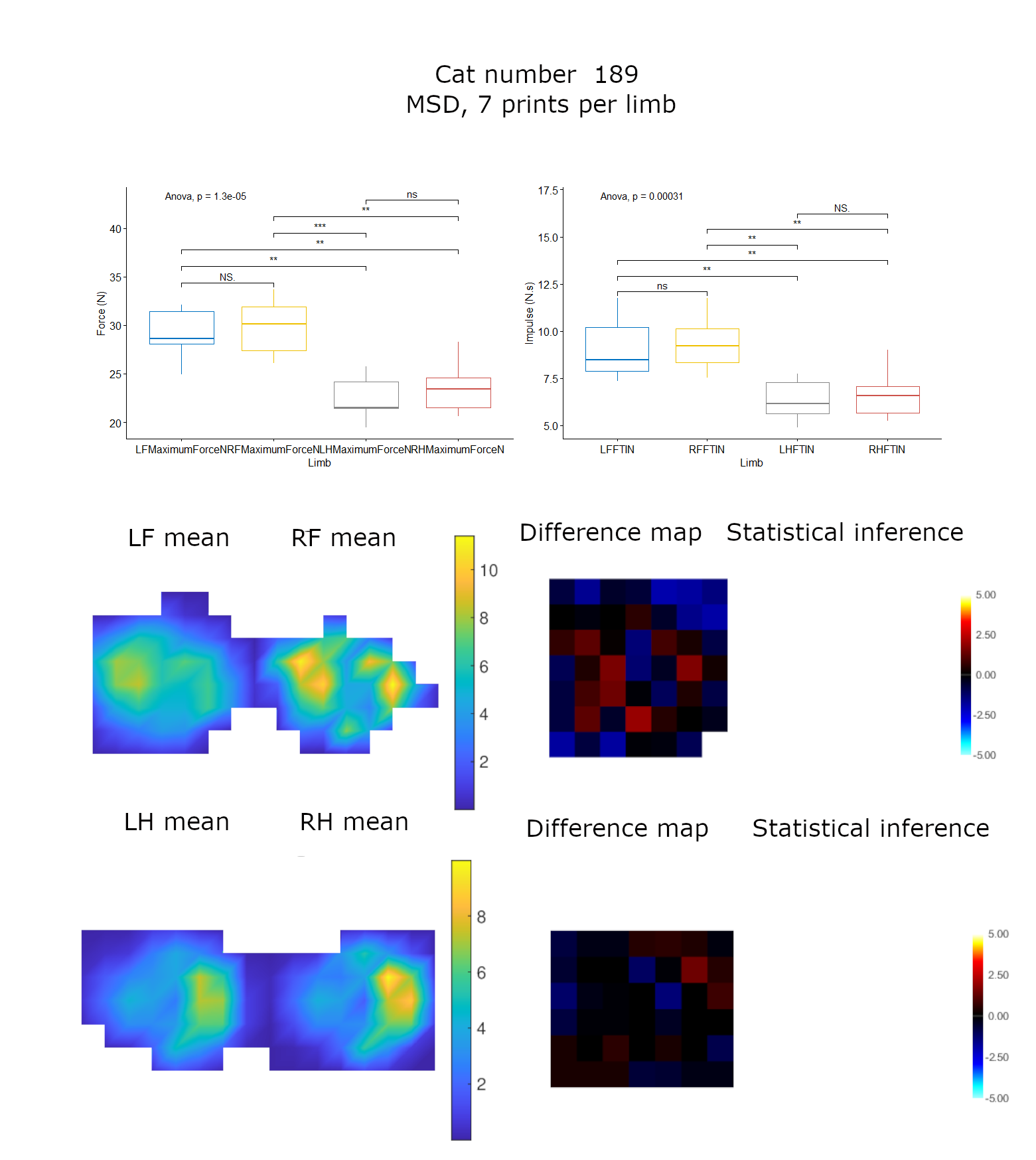

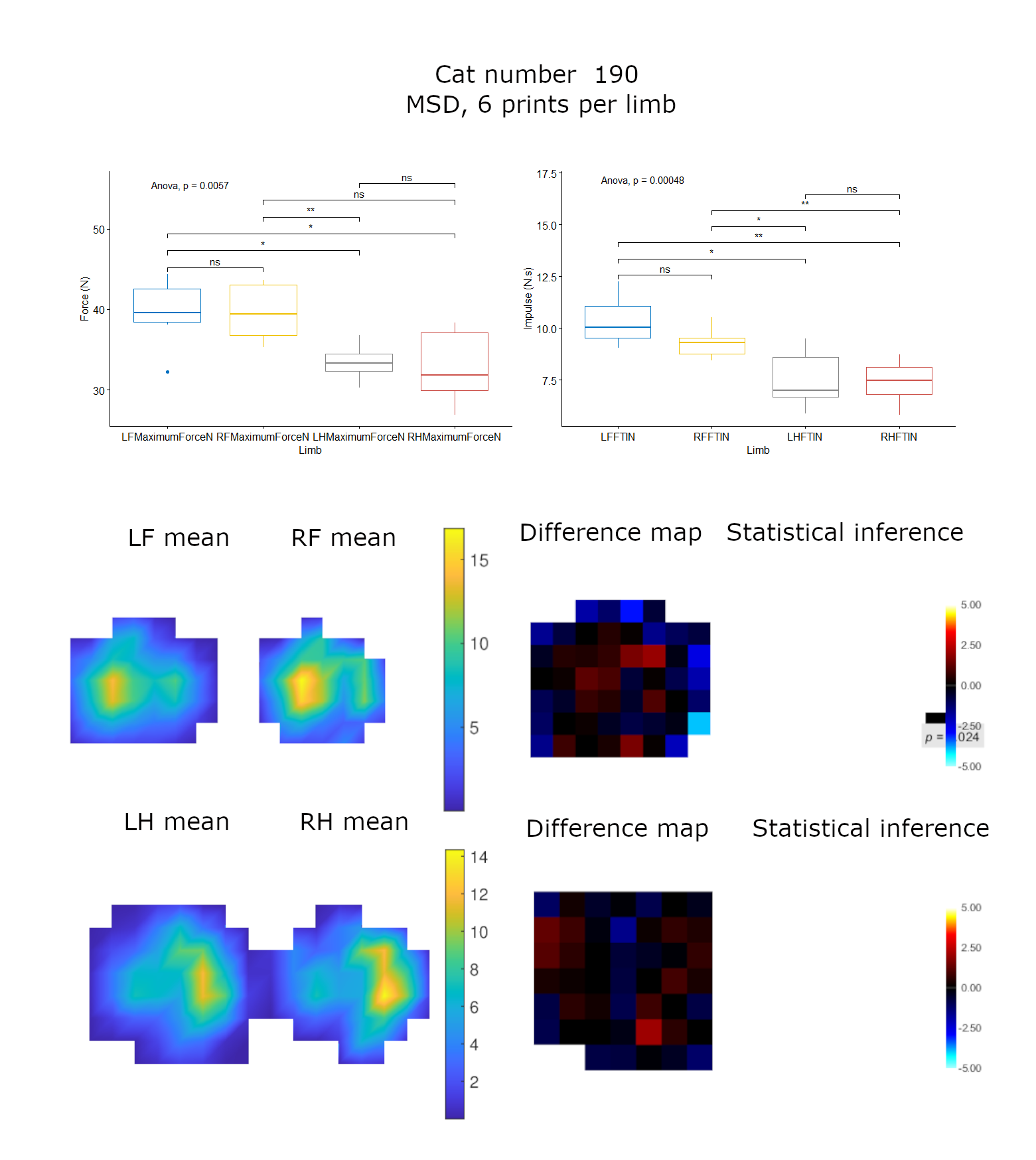

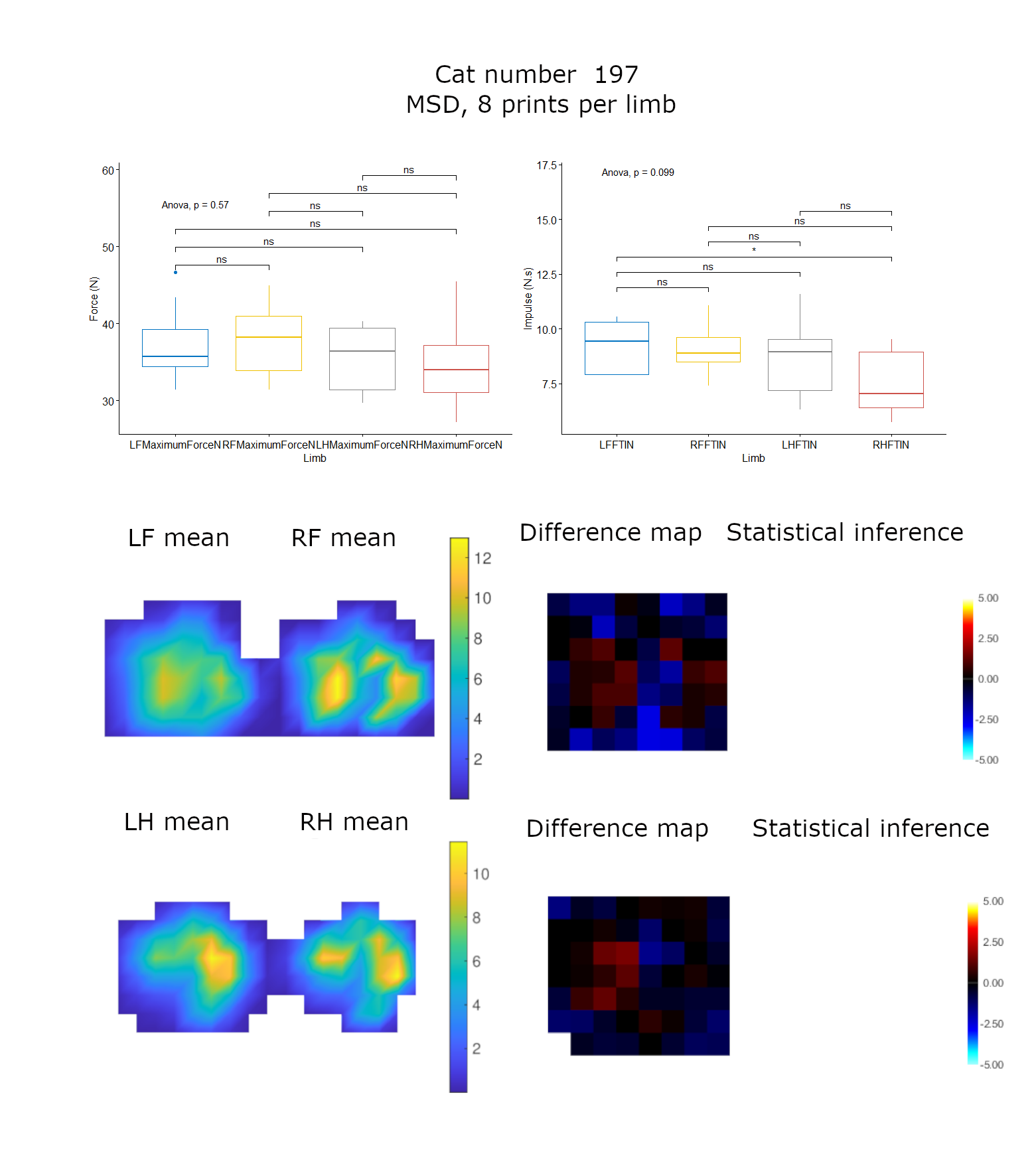

Supplement: S1 File — (DOCX) [file pone.0314629.s003.docx]
