# Supplementary material for: Paw pressure and gait in middle-aged client-owned cats with and without naturally-occurring musculoskeletal disease
Source: PLoS One. 2024 Dec 18;19(12):e0314629. doi: 10.1371/journal.pone.0314629 (PMC11654939; doi:10.1371/journal.pone.0314629)
Supplement: Attachment — Submitted filename: Response to reviewers.docx [file pone.0314629.s005.docx]

Dear editorial team and reviewers,

Thank you for taking the time to review our submission ‘Paw pressure and gait in middle-aged client-owned cats with and without naturally-occurring musculoskeletal disease’ and your helpful comments which we have hopefully addressed below.

**Journal Requirements**

1. When submitting your revision, we need you to address these additional requirements.

Manuscript has been edited to comply with PLOS ONE Style requirements and figures have been checked using the PACE tool. .

2. Competing Interests section: We can confirm that this does not alter our adherence to PLOS ONE policies on sharing data and materials. Our competing interest section has been updated in the manuscript as below.

*‘AJG is an employee of the University of Liverpool, but his post is financially supported by Royal Canin, a division of Mars Petcare. AJG has also received financial remuneration for providing educational material, speaking at conferences, and consultancy work from this company; all such remuneration has been for projects unrelated to the work reported in this manuscript. At the time the study was performed, ND was undertaking a post-graduate studentship funded by Royal Canin. Since October 2020, ND has been employed by International Cat Care, but also holds a part-time post-doctoral research position at the University of Liverpool, funded by Royal Canin. This does not alter our adherence to PLOS ONE policies on sharing data and materials.’*

**Reviewer one**

Thank you for your helpful review, we hope are following amendments are satisfactory.

Point 1: Apologies, I had forgotten to apply the absolute value to the formular, this has now been corrected.

Point 2: Thank you for raising this point. I have clarified the groups a little more in the results (see below) and in doing so noticed a mistake in the main manuscript, one of the forelimb cases had been miss counted as being multilimb, this mistake has been corrected. This mistake did not affect the files being used for statistical analysis and will have no impact on the results.
*‘Thirteen (59%) of the 22 cats with MSD had multiple limb involvement (both fore and hindlimb involvement), 7 (32%) had forelimb involvement (2 cats unilateral RF, 2 cats bilateral and 3 cats unilateral LF) and 2 (9%) had bilateral hindlimb involvement.’*

Kind regards,

Dr Nathalie Dowgray

University of Liverpool
