# Supplementary material for: Paw pressure and gait in middle-aged client-owned cats with and without naturally-occurring musculoskeletal disease
Source: PLoS One. 2024 Dec 18;19(12):e0314629. doi: 10.1371/journal.pone.0314629 (PMC11654939; doi:10.1371/journal.pone.0314629)
Supplement: Attachment — Submitted filename: Cover letter Revision 2.docx [file pone.0314629.s006.docx]

Dear Editor and Reviewer 2,

Thank you for your time and thoughts in reviewing our paper ‘Paw pressure and gait in middle-aged client-owned cats with and without naturally-occurring musculoskeletal disease’. Your comments and advice are very much appreciated. We have carefully considered all points made and made amendments to the manuscript as a result. These changes, and our responses to your comments, are covered in the point-by-point response that follows:

**1.** *The authors should consider to redistribute the animals into experimental groups more carefully. Based upon the number and position of the affected limb(s) well-defined groups should be created and each of these groups should be compared to the intact data. This is important because with locomotor diseases compensational processes can strongly distort the collected data depending on the number and sides of the affected limb or limbs. This way the mentioned “intra-cat and intercat” gait variabilities may be avoided, and there would be a chance to detect significant differences in some of the parameters even if “n” of the groups obviously drops this way. Please make clear what groups and subgroups you intend to set up and what plans you intend to make in order to find possible significance between these groups.*

We have taken into consideration comments about regrouping the cats and further analysis. The sub-setting the cats with musculoskeletal disease into different groups was something we had looked at previously when analyzing the data, but had deliberately excluded as the group sizes tend to be very small. Given the variation in our data (discussed at length in our manuscript), we felt it somewhat redundant to include such small group comparisons. However, as requested, we have now included aspects of this in our resubmission please see, lines 119-120 and 361-373 of our resubmission. The only significance found, at this level between the sub-groups, was that diseased forelimbs had significantly greater forelimb maximum PVF than non-diseased forelimbs; however, this significance disappeared when comparing percentage of total force (to account to some extent for size differences) or when bodyweight was added to the model creating a better fit by removing the MSD groupings.

**2.** *In the Discussion the authors should emphasize more the possible use of other gait measuring methods.*

Thank you for this comment. We have added additional detail in the discussion to include more detail on possible gait measuring methods. Please see lines 431-445 and lines 476 – 486

**3.** *line 419: Control of the velocity is not necessarily needed if the collected intact data is sorted and characterized by that aspect. By comparing the matching MSD data, different velocities make the assessment even more precise*.

Further discussion, on the utility of velocity in assessing musculoskeletal disease in cats, has been added in lines 454-455 and 482-485.

Thank you again for taking the time to review our paper. We hope this resubmission meets the required suggestions from this review process.

Yours sincerely,

Dr Nathalie Dowgray and co-authors
